# Stiff and self-healing hydrogels by polymer entanglements in co-planar nanoconfinement

**DOI:** 10.1038/s41563-025-02146-5

**Published:** 2025-03-07

**Authors:** Chen Liang, Volodymyr Dudko, Olena Khoruzhenko, Xiaodan Hong, Zhong-Peng Lv, Isabell Tunn, Muhammad Umer, Jaakko V. I. Timonen, Markus B. Linder, Josef Breu, Olli Ikkala, Hang Zhang

**Affiliations:** 1 Department of Applied Physics, Aalto University, Espoo, Finland; 2 Center of Excellence in Life-Inspired Hybrid Materials (LIBER), Aalto University, Espoo, Finland; 3 Bavarian Polymer Institute and Department of Chemistry, University of Bayreuth, Bayreuth, Germany; 4 Department of Bioproducts and Biosystems, School of Chemical Engineering, Aalto University, Espoo, Finland

**Keywords:** Soft materials, Nanoscale materials

## Abstract

Many biological tissues are mechanically strong and stiff but can still heal from damage. By contrast, synthetic hydrogels have not shown comparable combinations of properties, as current stiffening approaches inevitably suppress the required chain/bond dynamics for self-healing. Here we show a stiff and self-healing hydrogel with a modulus of 50 MPa and tensile strength up to 4.2 MPa by polymer entanglements in co-planar nanoconfinement. This is realized by polymerizing a highly concentrated monomer solution within a scaffold of fully delaminated synthetic hectorite nanosheets, shear oriented into a macroscopic monodomain. The resultant physical gels show self-healing efficiency up to 100% despite the high modulus, and high adhesion shear strength on a broad range of substrates. This nanoconfinement approach allows the incorporation of novel functionalities by embedding colloidal materials such as MXenes and can be generalized to other polymers and solvents to fabricate stiff and self-healing gels for soft robotics, additive manufacturing and biomedical applications.

## Abstract (web summary)

Mechanical stiffness and self-healing properties are difficult to combine in synthetic hydrogels. Using polymer entanglements in co-planar nanoconfinement, stiff and self-healing hydrogels are fabricated, with applications in biology and engineering.

## Main

Biological tissues possess extraordinary properties unparalleled by synthetic materials, such as self-repair, adaptation, and intricately balanced mechanical properties between stiffness, strength and toughness. Prominent examples include the remodelling of bones after damage^1^, strengthening of muscles after cyclic stresses^2^ and self-repair of strong tissues^1^. Considered as the synthetic equivalent of biological tissues, hydrogels provide unique characteristics^3–9^ such as water and nutrient transport capability, excellent biocompatibility, ionic conductivity and bioinspired properties, advantages that are generally limited in conventional elastomer systems.

Self-healing soft materials promise great potential in, for example, artificial skins, soft robotics and biomedical applications^10–12^. Self-healing hydrogels are typically based on dynamically exchangeable molecular interactions in the polymer networks, such as hydrogen bonds^13^, hydrophobic interactions^14^, physical adsorption^15^, host–guest interactions^16^, electrostatic interactions^17,18^, dynamic covalent bonds^19^ or combinations of them^20^. Although most self-healing hydrogels have reported low Young’s moduli below 100 kPa, some systems have demonstrated enhanced Young’s moduli between 4 MPa and 10 MPa (refs. ^18,21–23^). In addition, stiff hydrogels with modulus higher than 100 MPa have been reported in, for example, Ca^2+^-crosslinked^24^, supramolecular^25^ and mineralized^26^ hydrogels. Yet, they do not possess the ability to self-heal, as the used mechanisms for high stiffness hinder dynamic bond exchange and chain diffusions. By contrast, biological tissues such as human skin can reach elastic modulus of tens of megapascals^27^, yet possess remarkable self-repair capabilities. Therefore, there exists a knowledge gap in artificial hydrogels to combine self-healing and high stiffness.

Nanoconfinement has demonstrated emerging properties not achievable in bulk materials^28–30^, such as enhanced stretchability^31,32^, improved fracture strain and toughness^33,34^, and stiffening of rubbery response^35^. Furthermore, chain entanglements provide classic approaches to increase the toughness and strength of polymers^36^ and hydrogels^37^. Here we propose a general approach to stiffen hydrogels with high self-healing efficiency. This is achieved by constructing co-planar (slit-type) nanoconfinements using hectorite nanosheets, combined with in situ-formed highly entangled polymers. Such nanoconfinement leads to high stiffness above 50 MPa concomitantly with excellent self-healing capability, not yet reported in synthetic hydrogels.

## Highly entangled polymer network in co-planar nanoconfinement

**Fig. 1:**
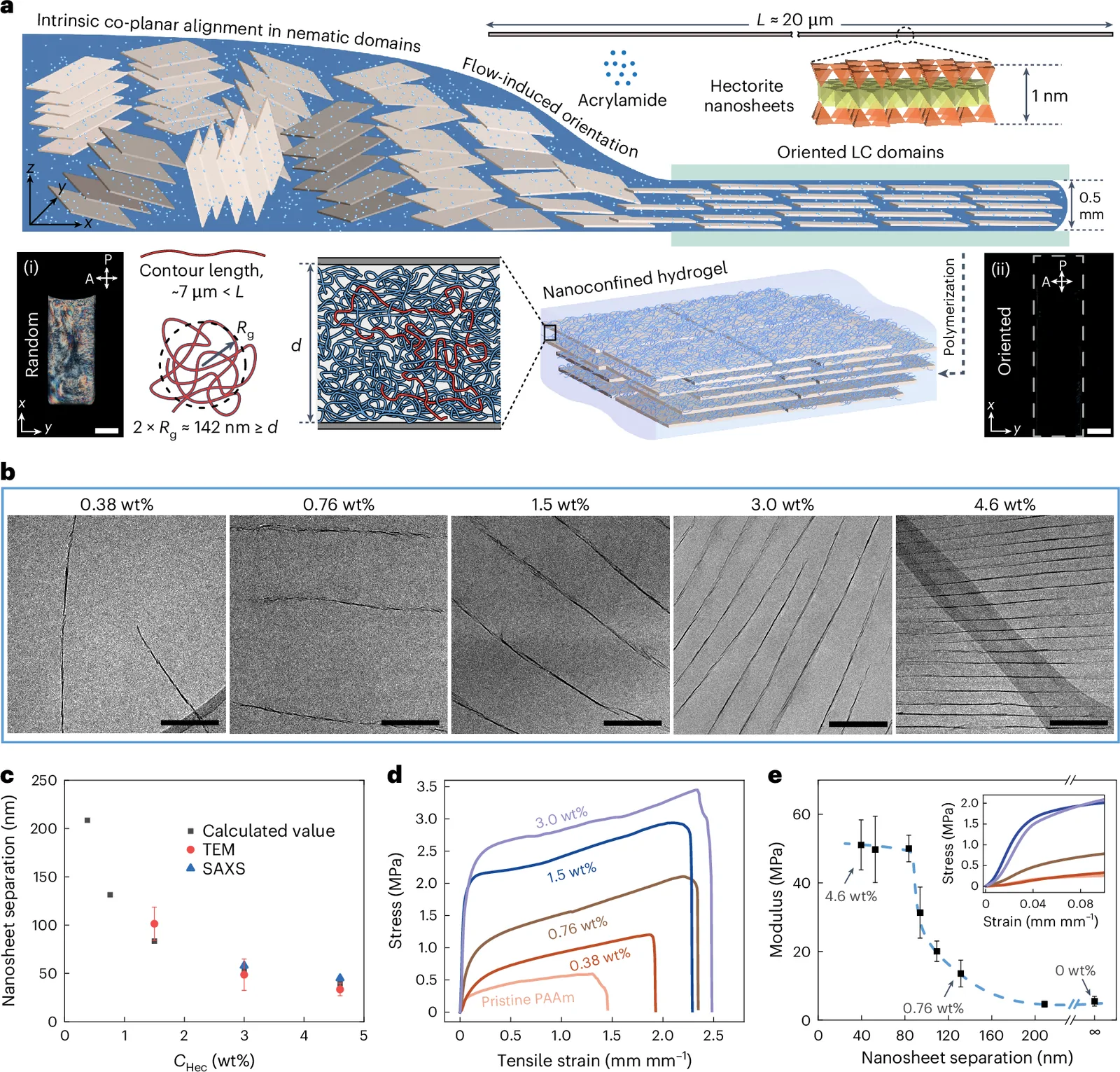
Nanoconfined hydrogels based on PAAm polymer entanglements within co-planar monodomain LC nanosheet scaffold. **a**, Schematic of the flow-induced orientation of nematic LC domains of nanosheets and in situ-formed entangled hydrogel. The high-AR monolayer nanosheets allow the co-planar alignment of nanosheets and the macroscopic orientation of LC domains by mild shear flow. (i) Polarized photograph of a pristine 1.5 wt% hectorite dispersion (5 mm thick). (ii) Polarized photograph of a monodomain 1.5 wt% hectorite dispersion (0.5 mm thick). The dashed line marks the contour of dispersion. A and P indicate the directions of the analyser and polarizer. *R*_g_ is the radius of gyration of PAAm. Scale bars, 5 mm. **b**, TEM characterization of aligned co-planar nanosheets in the nanoconfined hydrogel. Scale bars, 100 nm. **c**, Measurement of nanosheet separation using TEM and SAXS compared with the calculated values as a function of hectorite concentrations (*C*_Hec_). The TEM data are presented as mean values ± standard deviations from 50 measurements. **d**,**e**, Tensile stress–strain curves (**d**) and elastic moduli (**e**) depending on the nanosheet separation defining the confinement. The moduli are presented as mean values ± standard deviations from five samples. The inset in **e** shows an enlarged view of **d**. The colour codes in the inset are the same as those in **d**. Source data

**Extended Data Fig. 1:**
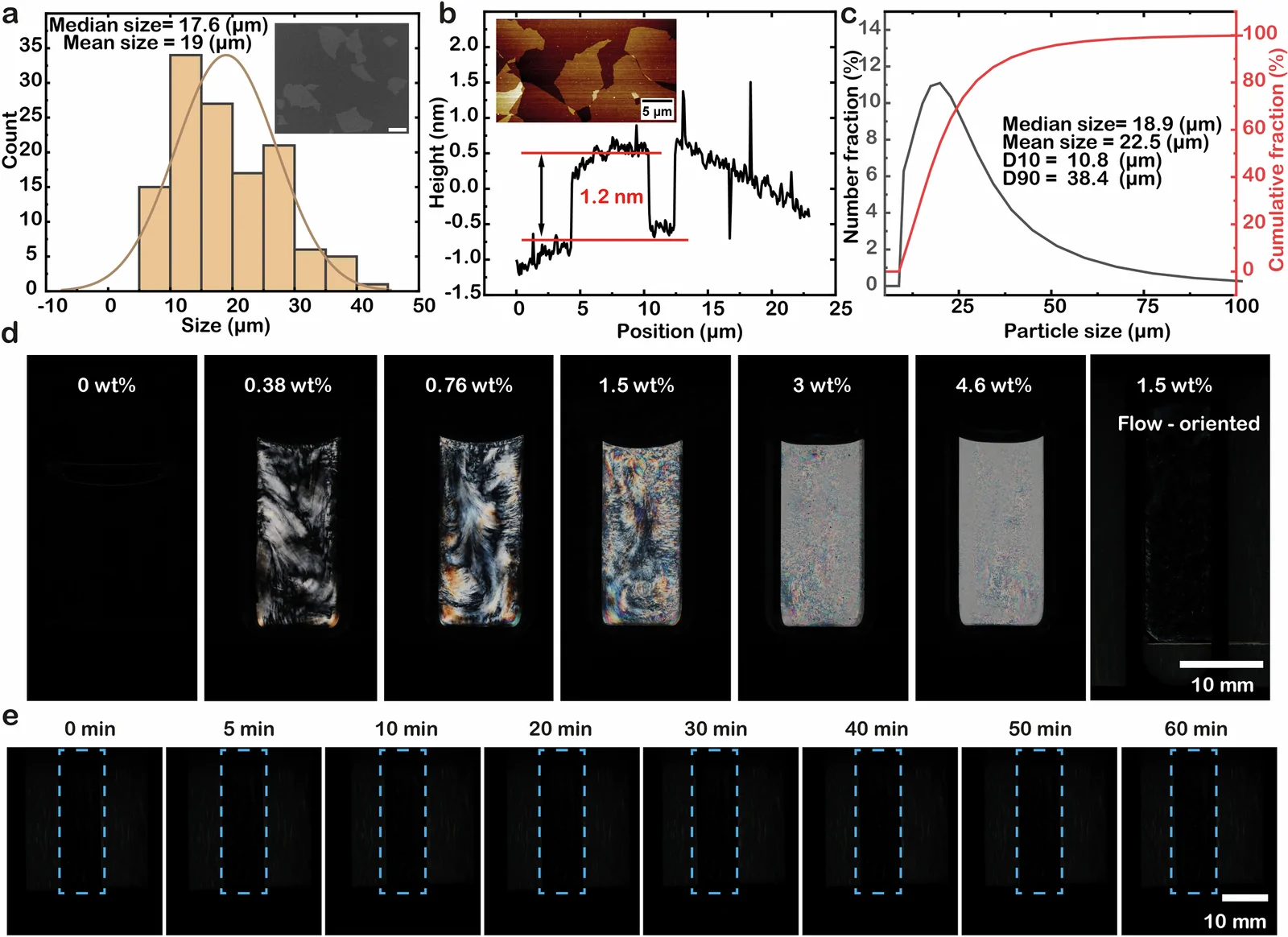
Characterization of synthetic hectorite. **a**, SEM image of hectorite nanosheets and their corresponding size distribution. Scale bar: 10 µm. Data are presented from 126 measurements. **b**, AFM image of hectorite nanosheets with a height of about 1.2 nm. **c**, SLS measurement of the size distribution of hectorite dispersion. **d**, Photos under polarized light of hectorite dispersions at different concentrations. The dispersions had a thickness of 5 mm, while the flow-oriented dispersion had a thickness of 0.5 mm. **e**, Stability of shear-oriented 1.5 wt% hectorite dispersion under polarized light with time. Dashed frames mark the position of the hectorite dispersion.

**Extended Data Fig. 2:**
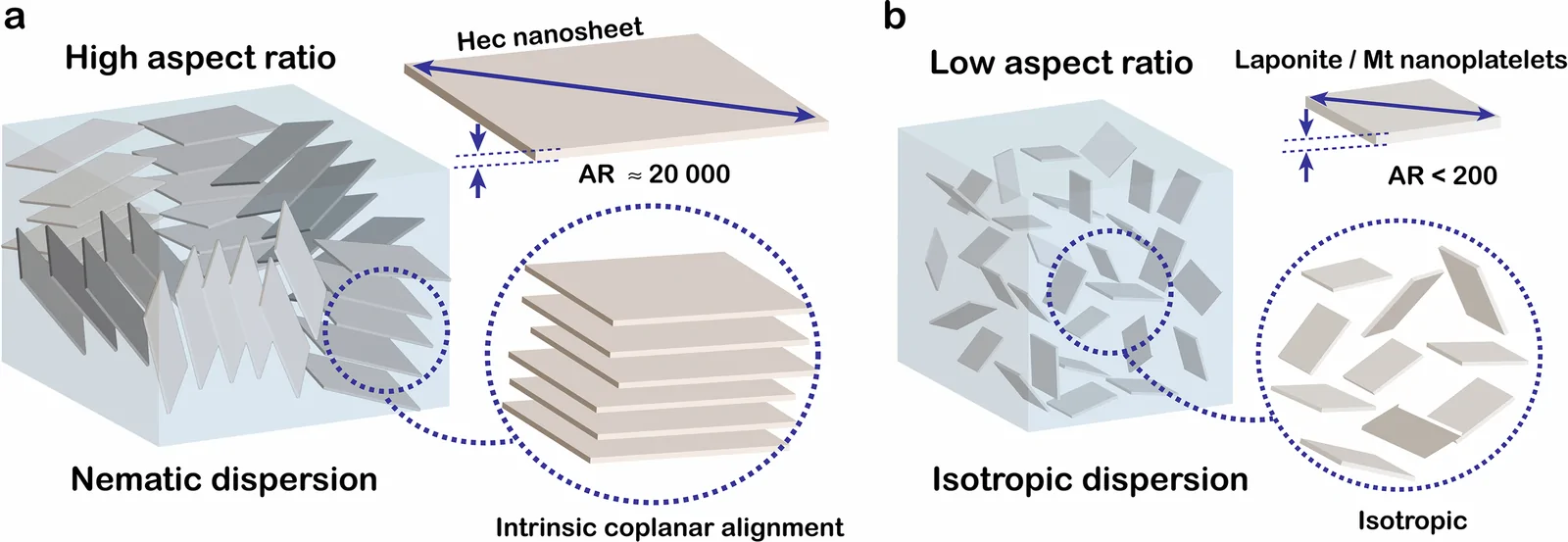
Comparison of dispersions of high aspect-ratio Hec nanosheets and low aspect-ratio nanoplatelets. **a**, Illustration of the nematic dispersion of Hec. **b**, Illustration of conventionally employed isotropic dispersion of low aspect ratio nanoplatelets.

The preparation of nanoconfined hydrogels is schematically shown in Fig. 1a. The thickness of the melt-synthesized hectorite nanosheet (Hec; [Na_0.48_]^inter^[Mg_2.57_ Li_0.47_]^oct^[Si_4_]^tet^O_10_F_2_) (ref. ^38^) is 1 nm and the average diameter is 20 µm (Extended Data Fig. 1), resulting in an aspect ratio (AR) of around 20,000. The homogeneously charged clay nanosheets are spontaneously separated by one-dimensional dissolution in water to uniform separations above 50 nm (ref. ^39^), controlled by their concentrations^40^. The strictly monolayer anionic nanosheets are held in co-planar alignment by electrostatic repulsion, which does not require external fields in contrast to conventionally used high fields for achieving alignment in nanocomposite hydrogels^15^^,^^41^^,^^42^. As the diameter of the nanosheets is orders of magnitude larger than the separation, rotation is hindered, which leads to a nematic phase even at low concentrations (<0.1 vol%). The individual liquid-crystalline (LC) domains are statistically oriented as indicated by the birefringence (Fig. 1a(i) and Extended Data Fig. 1), whereas suspensions of natural clays such as montmorillonite (Mt) are isotropic at these concentrations due to their low AR below 200 (refs. ^43^^,^^44^; Extended Data Fig. 2).

The nematic domains can be readily oriented and merged into a stable and oriented lamellar monodomain extending over at least a few centimetres by mild shear-induced flow (Extended Data Fig. 1 and Supplementary Fig. 1). The polarized image of the dispersion after shear flow in Fig. 1a(ii) shows no birefringence in the *x*–*y* plane, demonstrating the parallel orientation of the monodomain to this plane. The nanoconfined hydrogel is then formed by ultraviolet (UV)-initiated radical polymerization of the acrylamide, which results in a physically entangled polyacrylamide (PAAm; 62 wt% aq.; Supplementary Fig. 2) network within hectorite nanoconfinement, denoted as nanoconfined Hec-PAAm hydrogel.

**Extended Data Fig. 3:**
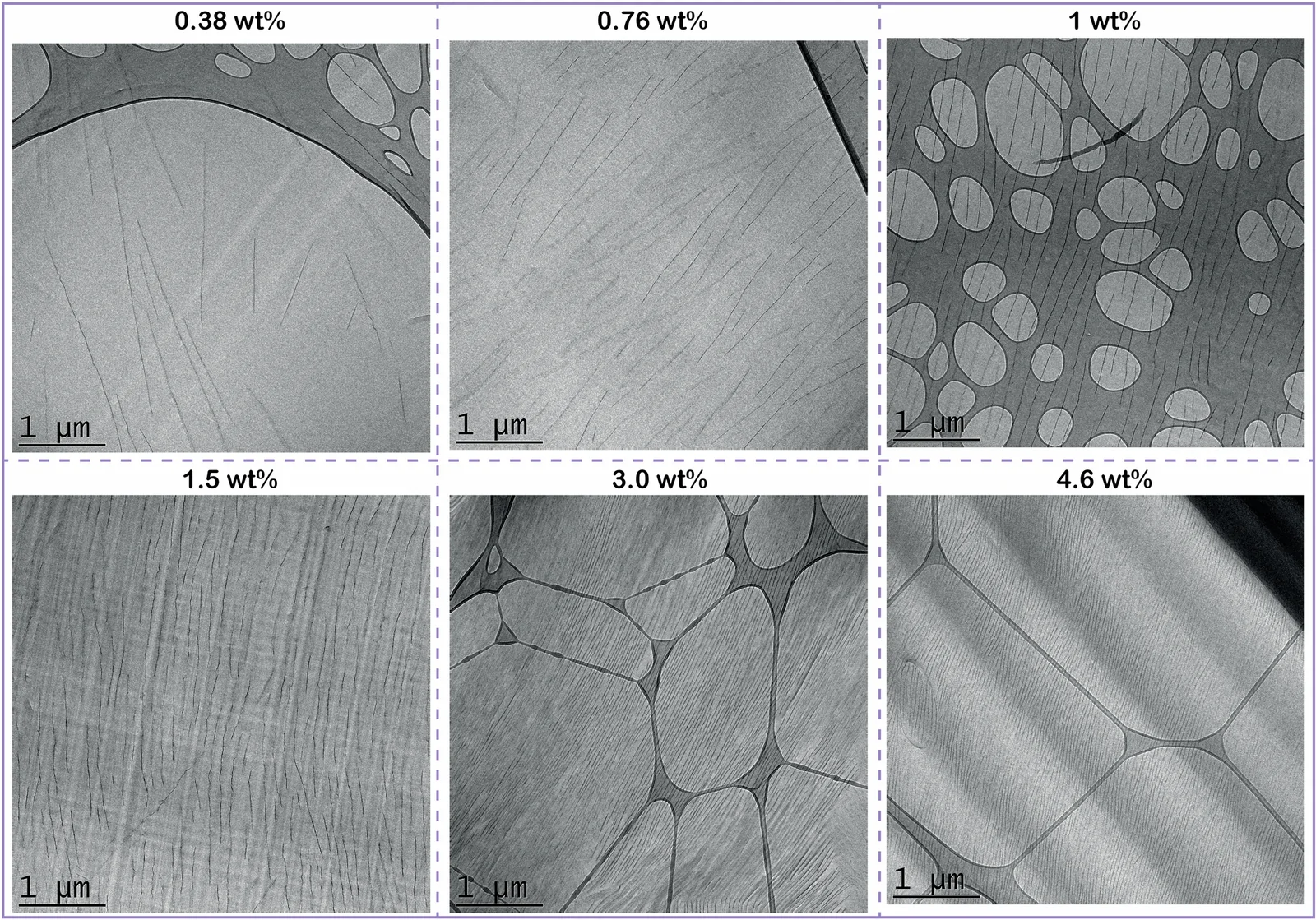
Cryo-TEM images of nanoconfined Hec-PAAm hydrogels at low magnification. The hydrogels contained different hectorite concentrations from 0.38 wt% to 4.6 wt%.

**Extended Data Fig. 4:**
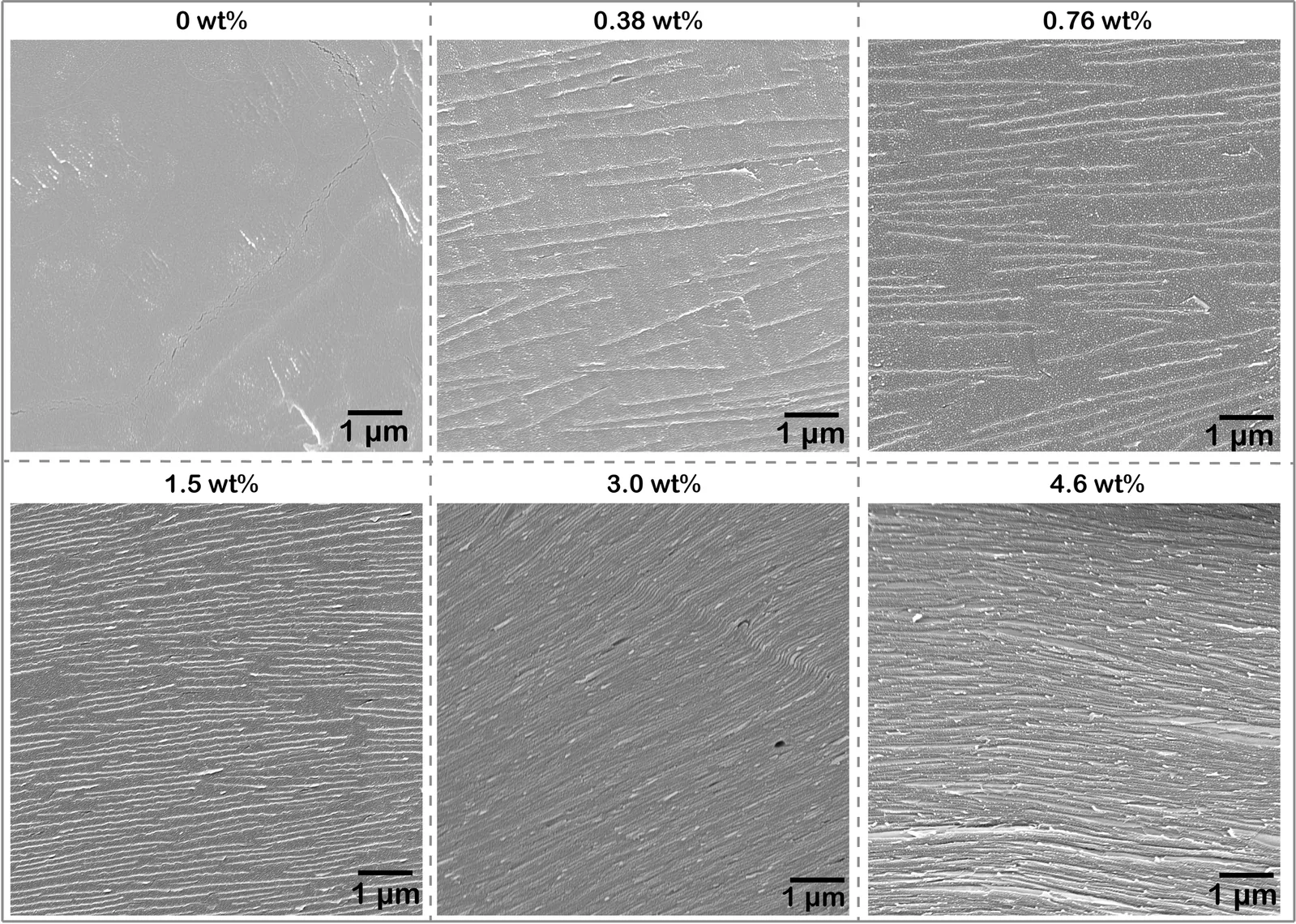
SEM images of the cross-sections of nanoconfined Hec-PAAm hydrogels. The hydrogels contained different hectorite concentrations from 0 wt% to 4.6 wt%.

The separation between nanosheets at different hectorite concentrations (*C*_Hec_) has been characterized by cryo-transmission electron microscopy (cryo-TEM) and small-angle X-ray scattering (SAXS) (Fig. 1b,c and Supplementary Fig. 3). The long-range uniform co-planar alignment of hectorite nanosheets is further corroborated by TEM and scanning electron microscopy (SEM) characterizations (Extended Data Figs. 3 and 4, respectively). The measured nanosheet separations match well with the calculated^40^ values of strictly monolayer dispersions (Fig. 1c), confirming the full delamination of nanosheets without aggregation. For *C*_Hec_ below 1 wt%, the separation is larger than 100 nm, which results in less co-planar alignment due to increased—though still limited—rotational freedom between the nanosheets, whereas the separation becomes highly uniform for concentrations above 1.5 wt% (Fig. 1b,c). The large separation, high polymer content and ultrahigh AR of the nanosheets distinguish the nanoconfined hydrogels from previous nacre-like materials^45–47^.

The uniaxial tensile curves of the hydrogels containing different *C*_Hec_ values are summarized in Fig. 1d and Supplementary Fig. 4, which show a characteristic two-stage behaviour. The first stage at small strain (<3%) is marked by a high Young’s modulus (*E*) of tens of megapascals, where the tensile stress increases to a few megapascals. The second stage shows plastic deformations with slight strain hardening and large elongations up to a strain of 2.5 for 3.0 wt% hectorite and an ultimate tensile strength (UTS) of up to 4.2 MPa for 4.6 wt% hectorite (Supplementary Fig. 4). At *C*_Hec_ = 0.38 wt%, *E* is only 4.7 MPa, similar to the PAAm gel without hectorite (5.5 MPa). There exists a drastic increase in *E* as the nanosheet separation decreases, reaching 50 MPa at 83.5 nm (corresponding to 1.5 wt%; Fig. 1e), that is, a tenfold increase compared with hydrogels containing 0.38 wt% hectorite with separations above 209 nm. We attribute this pronounced change to the oriented nanoconfinement (Supplementary Fig. 5 shows the tensile test of non-oriented Hec-PAAm hydrogel), when the diameter of individual PAAm chains (~142 nm) approaches the dimension of co-planar nanoconfinement (Fig. 1e). The diameter is estimated from the radius of gyration (*R*_g_) of PAAm chains of around 71 nm (ref. ^48^), based on the weight average molecular weight $\bar{M}_{\rm{W}}$ of 1.64 × 10^6^. Such an abrupt transition in the modulus cannot be accounted for when using classical composite mechanical models, such as the Halpin–Tsai model^49^, which suggests a linear dependence of Young’s modulus on filler content (Supplementary Methods, Supplementary Table 1 and Supplementary Fig. 6). Furthermore, the influence of strain rate and water content on the mechanical properties of Hec-PAAm hydrogels is provided in Supplementary Figs. 7–9. The increasing stiffness on dehydration agrees with previous reports on hydrogels containing lamellar bilayers^50^.

**Fig. 2:**
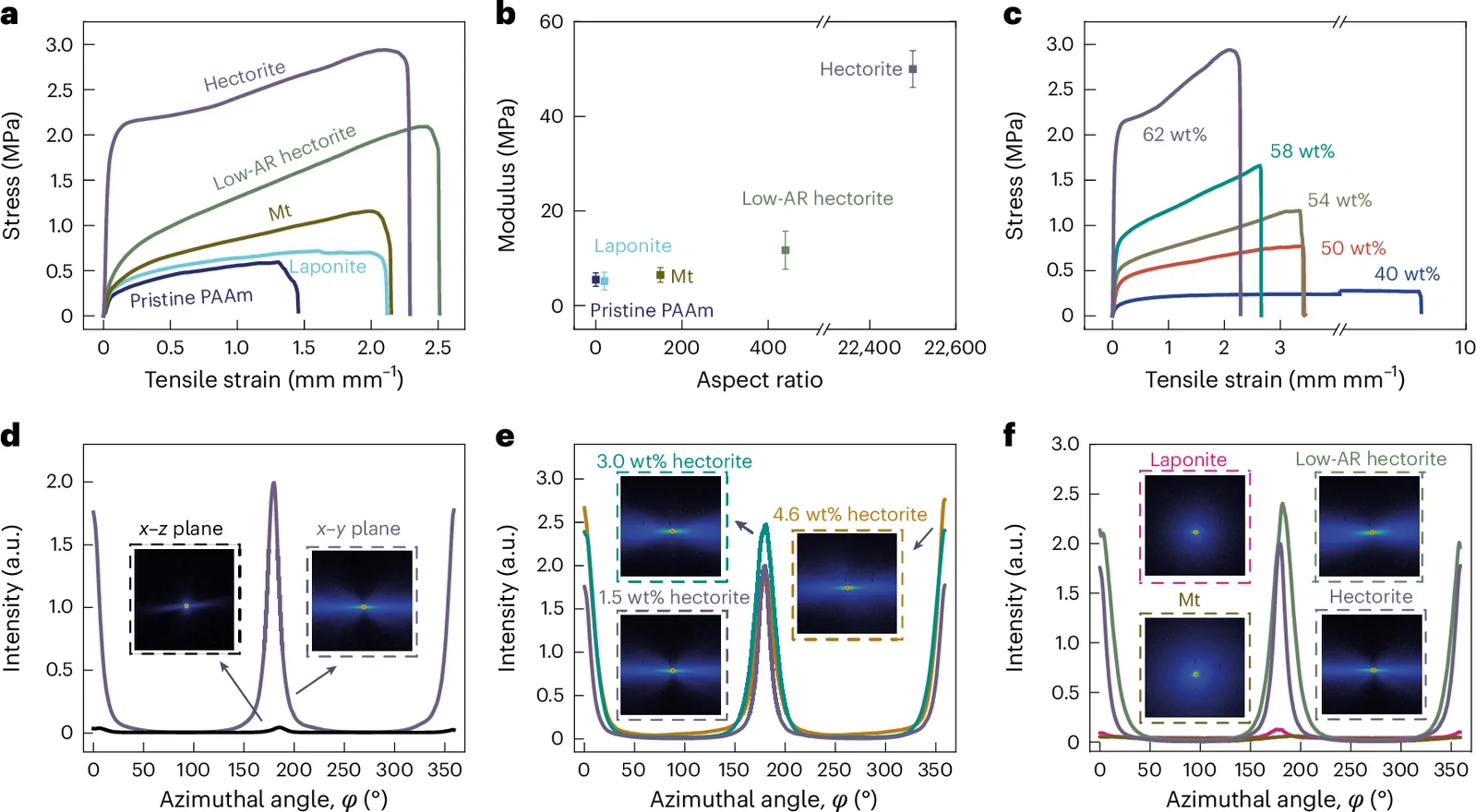
Effect of clay nanoplatelet/nanosheet AR and polymer concentration. **a**, Comparison of hydrogels prepared with nanoplatelets/nanosheets with varying ARs. Only high-AR hectorite nanosheets lead to substantially enhanced stiffness and strength. **b**, Dependence of elastic moduli on the AR of nanoplatelets or nanosheets. The moduli are presented as mean values ± standard deviations from five samples. **c**, Critical role of polymer concentration to promote nanoconfined entanglement for enhanced stiffness and strength. **d**, SAXS data in the *x*–*y* plane and *x*–*z* plane proving the in-plane alignment of nanosheets in the nanocomposite hydrogel. **e**, High anisotropy by shear-induced alignment in nanoconfined hydrogels for different hectorite concentrations. **f**, Weaker alignment due to decreasing AR evidenced from the SAXS data of hydrogels containing different nanoclays. Unless otherwise stated, all hydrogels contained 1.5 wt% nanoplatelets/nanosheets and 62 wt% PAAm. Source data

Both nanoconfinement and polymer entanglement are vital for achieving high stiffness (Fig. 2). Figure 2a,b depicts the effect of AR of nanoplatelets/nanosheets on the tensile behaviour at a constant polymer concentration of 62 wt%. Pronounced stiffening and strengthening are observed only for the high-AR hectorite (AR ≈ 20,000), whereas Laponite (AR ≈ 20) showed no noticeable effect on the mechanical properties (Fig. 2a). For nanosheets with ARs between these values, such as Mt (AR ≈ 150; Supplementary Fig. 10) or small (low-AR) hectorite (AR ≈ 440; Supplementary Fig. 11), intermediate UTS is obtained. The *E* values in these samples are much smaller than that of high-AR hectorite, such as 11.7 MPa in low-AR hectorite.

**Extended Data Fig. 5:**
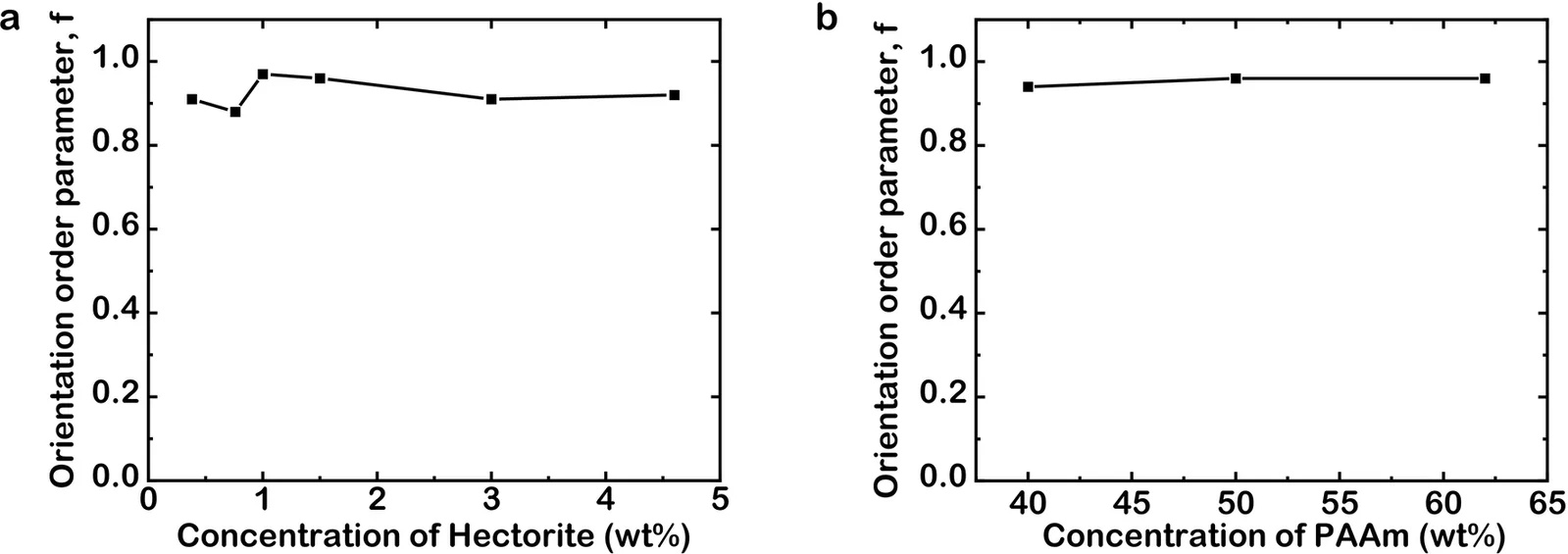
The orientation order parameters of Hec-PAAm hydrogels. **a**, Different concentrations of hectorite at 62 wt% PAAm. **b**, Different contents of PAAm at *C*_Hec_ = 1.5 wt%.

Additionally, high polymer concentration and strong entanglement are also crucial for high stiffness (Fig. 2c and Supplementary Fig. 12). Even a slight reduction in the PAAm concentration from 62 wt% to 58 wt% leads to a decrease in *E* from 50 MPa to 17 MPa, whereas the UTS value decreases from 2.9 MPa to 1.7 MPa. At 40 wt% PAAm, the nanoconfined hydrogel became soft and stretchy, allowing elongation up to a strain of 9.6 at a UTS of 0.3 MPa. The polymer concentrations did not affect the alignment of the nanosheets, as confirmed by TEM, SEM and SAXS measurements (Supplementary Figs. 13–15 and Extended Data Fig. 5). The molecular weight and, thus, the size of the polymer chains are also independent of the monomer concentrations (Supplementary Table 2). Therefore, the enhanced mechanical properties can be attributed to the high degree of polymer entanglement under nanoconfinement.

**Extended Data Fig. 6:**
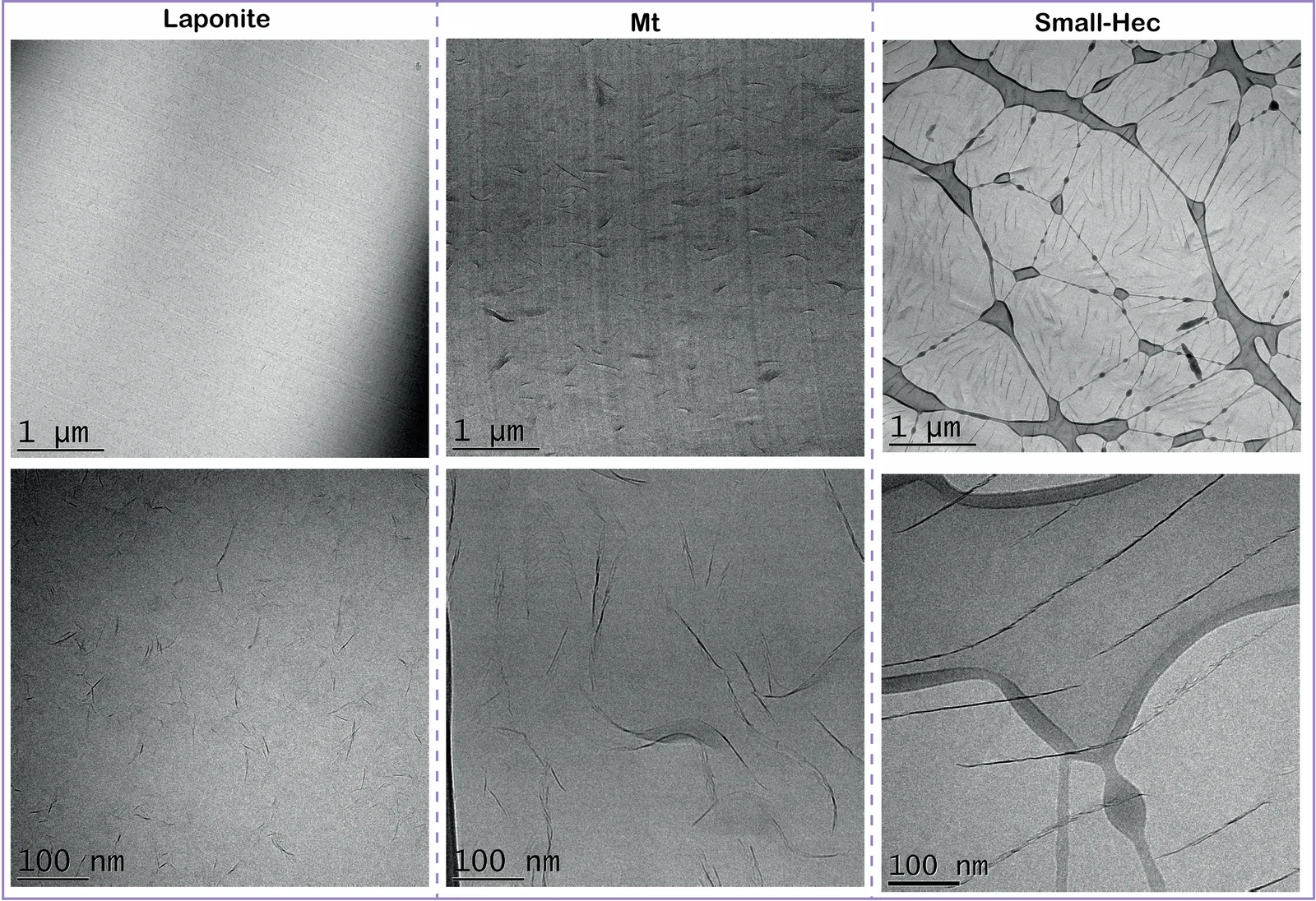
Cryo-TEM images of clay-PAAm hydrogels. The hydrogels contained different types of clay nanoplatelets, that is, Laponite, Mt, and small Hec.

The flow-induced orientation of nanosheets with varying ARs is further characterized by SAXS measurements (Fig. 2d–f). In the *x*–*y* plane, that is, normal to the flow direction, weak anisotropy is observed at 1.5 wt% *C*_Hec_, which demonstrates predominate parallel alignment of the nanosheets to this plane. By contrast, the *x*–*z* plane shows pronounced anisotropy, for both 1.5 wt% (Fig. 2d) and higher *C*_Hec_ (Fig. 2e). This is also reflected in the orientation order parameters (Extended Data Fig. 5), which are above 0.91 for *C*_Hec_ higher than 1.5 wt%. For *C*_Hec_ below 1 wt%, weaker anisotropy has been observed, whereas the pristine PAAm hydrogel shows no anisotropy (Supplementary Fig. 16). Smaller nanoplatelets, such as Laponite and Mt, show rather isotropic SAXS patterns at 1.5 wt% in the *x*–*z* plane, whereas the small hectorite (AR, 440) shows weak alignment (Fig. 2f). The SAXS measurements are further corroborated by TEM characterizations (Extended Data Fig. 6).

## Efficient self-healing and strong adhesion

**Fig. 3:**
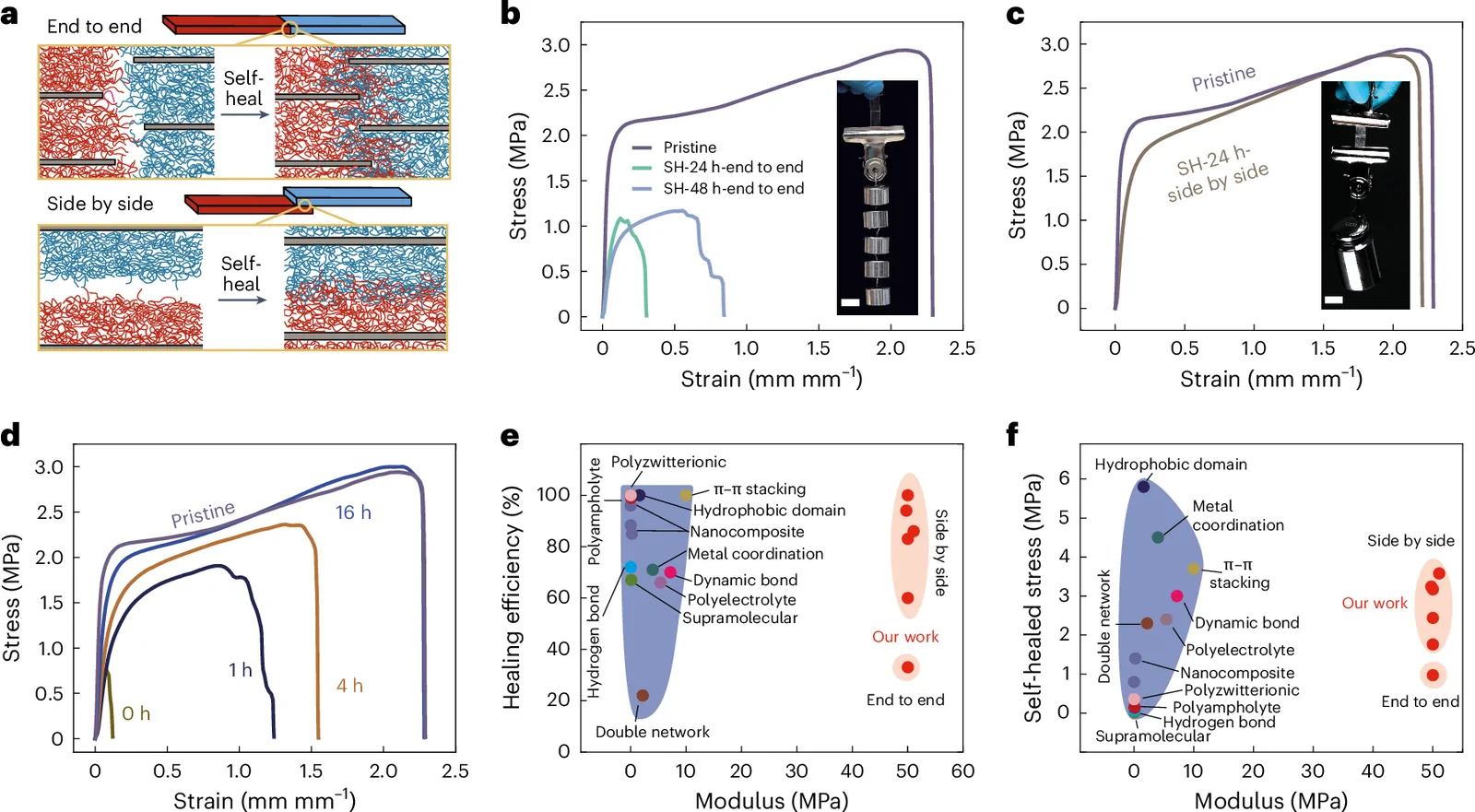
Self-healing based on polymer entanglements in co-planar nanoconfinement. **a**, Illustration of self-healing mechanisms based on polymer entanglements along different directions. **b**, Tensile curves of nanocomposite hydrogels self-healed (SH) in the *y*–*z* plane (end to end) for different durations. The inset shows the side view of a self-healed hydrogel ribbon (thickness, 0.5 mm) holding a weight of 250 g. Scale bar, 10 mm. **c**, Tensile curves of nanocomposite hydrogels self-healed in the *x*–*y* plane (side by side). The inset shows the front view of a self-healed hydrogel ribbon (thickness, 0.5 mm) holding a weight of 500 g. Scale bar, 10 mm. **d**, Kinetics of self-healing. **e**, Self-healing of nanoconfined hydrogels outperforms literature data in terms of Young’s modulus. Self-healing efficiencies obtained from different methods are indicated in the plot. The values are presented as mean values from five samples. **f**, Ultimate tensile stress of self-healed nanoconfined hydrogels outperforms reported self-healing hydrogels in terms of Young’s modulus. Source data

One prominent feature of the nanoconfined hydrogel is its ability to self-heal despite the high modulus (Fig. 3). Due to the high anisotropy of the hydrogel, we define self-healing in two directions: (1) end-to-end self-healing in the *y*–*z* plane and (2) side-by-side self-healing in the *x*–*y* plane (Fig. 3a). Though less commonly studied, side-by-side self-healing is highly advantageous for assembling the hydrogel thin films into complex three-dimensional geometries (see the ‘Nanoconfinement as a universal reinforcement strategy’ section). In the end-to-end configuration, the hydrogels were cut and then merged at the cross-section, after which the tensile measurement was performed after certain durations (Fig. 3b). As the uppermost layer of the polymer network is less than 100 nm thick, the hydrogel’s surface dries quickly in air even at a relative humidity of around 30%. To rehydrate the surface of the hydrogel for self-healing, we have applied a minor amount of water at the interface before reattaching the hydrogels. Here a recovery of UTS up to 1.2 MPa and a strain at failure of up to 0.8 after 48 h have been recorded (Fig. 3b). The average self-healing efficiency is around 33% measured by the UTS value (Supplementary Fig. 17). The self-healed interface is rather strong, as demonstrated by the sample with a thickness of only 0.5 mm withstanding a weight of 250 g (Fig. 3b, inset). Here the interface did not fully recover the original strength, possibly due to the high stiffness of the material that prevents conformal contact between the cut surfaces. Nevertheless, it is remarkable that self-healing took place in a highly stiff hydrogel without sophisticated designs of molecular interactions.

Furthermore, the side-by-side configuration allows up to 100% of self-healing efficiency measured by UTS and strain at failure (Fig. 3c). The two samples were brought into contact in a side-by-side manner with an overlapped length of 2 mm. For samples containing 1.5 wt% (Fig. 3c) and 3.0 wt% hectorite (Supplementary Fig. 18), the self-healing efficiency reached 94–100% after 24 h, corresponding to a self-healed UTS above 3 MPa. The self-healing with shorter overlapped length is shown in Supplementary Fig. 19, where 1 mm length led to a self-healing efficiency of 83% measured by the UTS value. The kinetics of side-by-side self-healing is shown in Fig. 3d, which shows a gradual increase in mechanical properties with time, and a self-healing efficiency of 60% can already be reached after 1 h. Tensile curves and side-by-side self-healing efficiencies under various healing times are provided in Supplementary Fig. 20. In addition, the Hec-PAAm hydrogel can also self-heal surface scratch damages in a similar timescale (Supplementary Fig. 21).

Self-healing in the Hec-PAAm hydrogel is based on the re-formation of PAAm polymer entanglements at the interface^51^. This is corroborated by the control experiment on pristine PAAm hydrogel without hectorite (Supplementary Fig. 22), where the PAAm hydrogel reached 92% self-healing after 24 h of contact. In addition, fluorescence recovery after photobleaching (FRAP) characterization of fluorescently labelled Hec-PAAm hydrogel reveals that the PAAm chains remain dynamic in nanoconfinement (Supplementary Figs. 23 and 24).

We further compare the self-healing performance of the nanoconfined hydrogels with previously reported hydrogels (Fig. 3e,f and Supplementary Table 3), which have shown self-healing between fully separated interfaces without extensive reprocessing (for example, by the melting of crystalline domains^52^). Conventionally, there exists a compromise between self-healing efficiency and the stiffness of the material. The Hec-PAAm system has made a substantial leap in the stiffness of the material by increasing the modulus to 50 MPa and maintaining a high self-healing efficiency between 33% and 100%. In terms of UTS of the self-healing hydrogels, our system is on par with the highest reported value at 3.6 MPa (Fig. 3f).

**Fig. 4:**
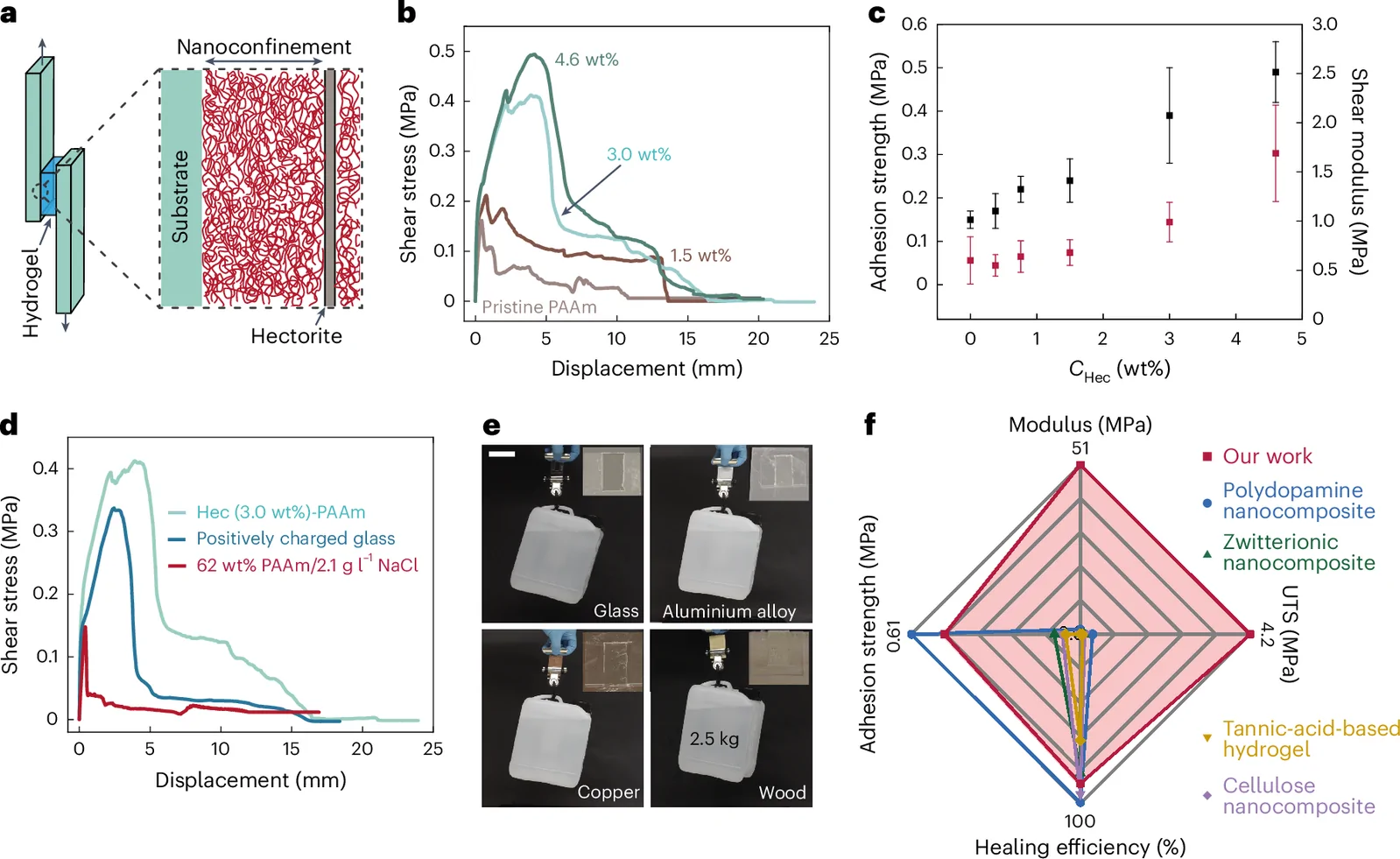
Strong adhesion in the nanoconfined Hec-PAAm hydrogels. **a**, Illustration of lap-shear adhesion test and nanoconfinement at the interface. **b**, Nanoconfinement leads to enhanced adhesion. **c**, Adhesion strength (black) and shear modulus (red) depending on the concentration of hectorite nanosheets for in situ-formed hydrogels. The values are presented as mean values ± standard deviations from five samples. **d**, Strong adhesion on positively and negatively charged surfaces, showing the universal effect of nanoconfinement. **e**, Photographs showing different substrates adhered by hydrogels of 2 cm^2^ and thickness of 0.5 mm holding a weight of 2.5 kg. Scale bar, 5 cm. **f**, Radar map showing superior Young’s modulus, UTS, self-healing efficiency and adhesion strength on glass in the nanoconfined hydrogel (4.6 wt% hectorite) compared with reported systems. Source data

In addition to the self-healing capability, the nanoconfinement principle also allows considerable enhancement in the adhesion properties to different substrates, as demonstrated by lap-shear measurements (Fig. 4 and Supplementary Fig. 25). The hydrogel was formed in situ between glass or other types of substrate. The polymer network in contact with the substrate is also under interfacial nanoconfinement formed between the substrate and hectorite nanosheets (Fig. 4a). It is expected that such interfacial confinement is of similar size as the nanoconfinement in the bulk of the hydrogel, due to the electrostatic repulsion between the surface and the nanosheets. In the absence of clay, the maximum adhesion only reached 0.15 MPa (Fig. 4b,c). With 4.6 wt% hectorite, the maximum adhesive strength reached 0.49 MPa. The adhesion strength for post-adhered hydrogels reaches a maximum adhesive strength of 0.09 MPa after 48 h (Supplementary Fig. 26). The shear moduli can be calculated from the low-strain range of the lap-shear measurements before detachment of the hydrogel, which give a shear modulus of 1.7 MPa for hydrogel containing 4.6 wt% hectorite (Fig. 4c).

To confirm that the improved adhesive properties are due to the nanoconfinement effect, we have performed control experiments (Fig. 4d), which exclude the effects of surface charges or counterions. The in situ-formed hydrogel can easily withstand a static load of 2.5 kg on different types of substrate such as aluminium, copper and birch wood (Fig. 4e). It is remarkable that such high adhesion is achieved without the use of sophisticated molecular mechanisms such as catechol chemistry^53^. By introducing covalent bonds with the PAAm network through the silanization of the glass surface, the adhesion strength is further enhanced to 1.1 MPa at 3.0 wt% hectorite (Supplementary Fig. 27). The nanoconfinement strategy, thus, promises implications in the interfacial engineering between soft and hard substrates.

The adhesive properties of the nanoconfined hydrogel containing 4.6 wt% hectorite together with its UTS, self-healing efficiency (Supplementary Fig. 18) and Young’s modulus are further compared with reported systems in the radar map shown in Fig. 4f. The complete reference list is provided in Supplementary Table 4. So far, most hydrogel systems lack one or two of the above-mentioned properties^54^, and the nanoconfined hydrogel provides one of the first examples that combines high UTS, stiffness, adhesion strength and self-healing efficiency.

## Nanoconfinement as a universal reinforcement strategy

**Fig. 5:**
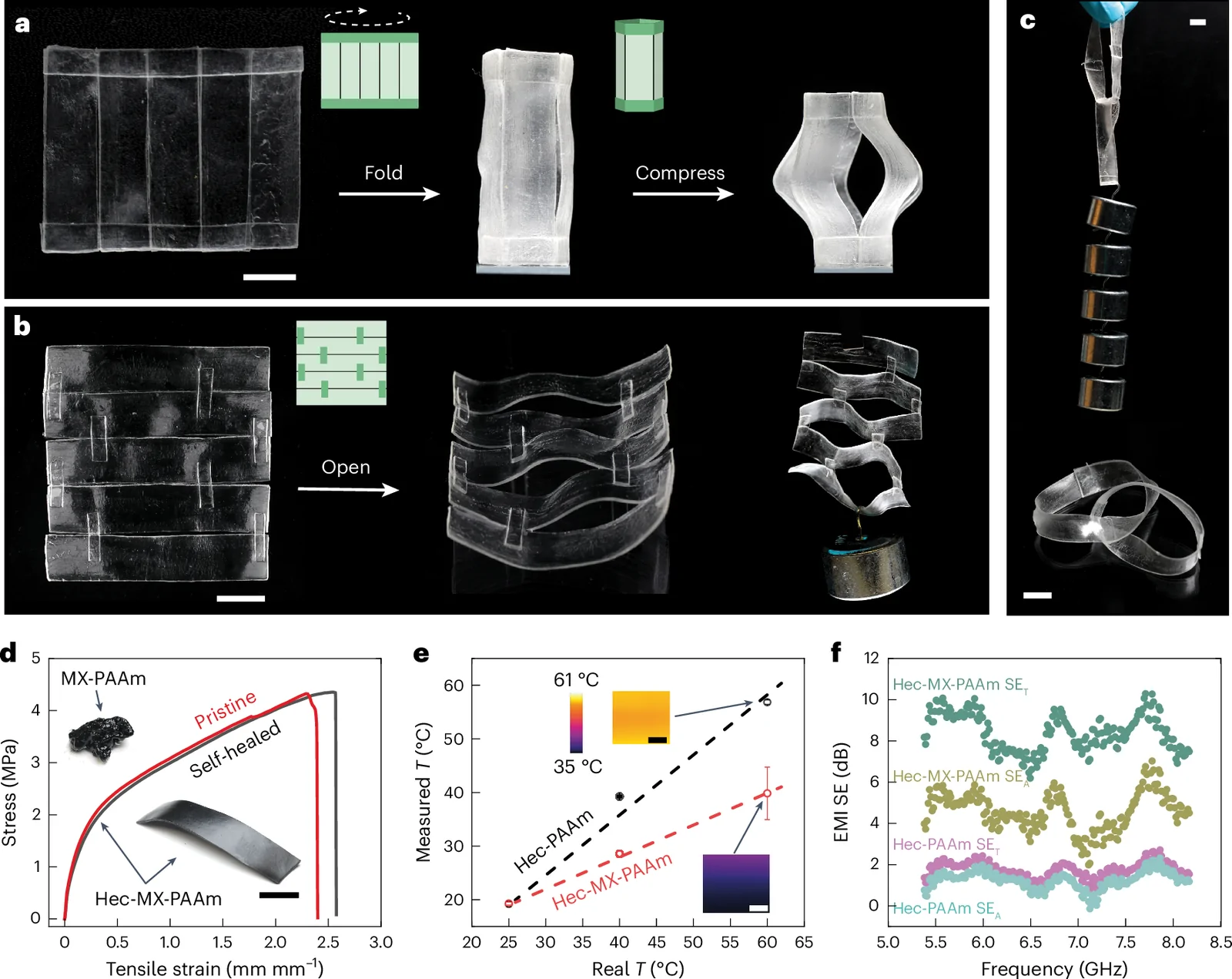
Demonstration of self-healing for assembled complex-shaped hydrogels and incorporation of new functionalities. **a**, Assembly of hydrogel ribbons into foldable lantern-like objects by self-healing. Scale bar, 1 cm. **b**, Kirigami-like assembly of hydrogel objects. Scale bar, 1 cm. **c**, Interlocked Möbius-ring-shaped hydrogels. The green area indicates the self-healed areas. Scale bar, 1 cm. **d**, Nanoconfined Hec-MX-PAAm hydrogel showing high stiffness and mechanical strength. The insets show the photographs of the Hec-MX-PAAm hydrogel and MX-PAAm hydrogel. Scale bar, 1 cm. **e**, Thermal camouflage of Hec-MX-PAAm hydrogel shown by the temperature measured by an IR camera. The insets show the IR images of the hydrogels at different temperatures. Scale bars, 1 mm. The values are presented as mean values ± standard deviations from five measurement points. The dashed lines are guides for the eyes. **f**, Enhanced EMI-shielding properties of the Hec-MX-PAAm hydrogel. SE, EMI-shielding effectiveness; SE_T_, total shielding effectiveness; SE_A_, absorption component of shielding effectiveness. The hectorite, MXene and PAAm concentrations are 1.5 wt%, 1.5 wt% and 62 wt%, respectively. Source data

The high stiffness and self-healing properties of nanoconfined hydrogels can be exploited to construct mechanically robust complex shapes. Rectangular hydrogel ribbons are healed together to form a three-dimensional lantern shape, which can be opened by compression or closed by stretching (Fig. 5a). Using the same ribbons and self-healing at dedicated locations, an array of hydrogels can be linked together to form a kirigami-like film, which opens under stretching (Fig. 5b). Moreover, Möbius-ring-shaped hydrogels can be formed by twisting the ribbon and letting the two ends heal, which would be difficult to fabricate via conventional hydrogel synthesis techniques. Figure 5c shows two interlinked hydrogel Möbius rings, which can withstand 250 g of weight. These examples illustrate the application potential of nanoconfinement for soft material fabrication.

**Extended Data Fig. 7:**
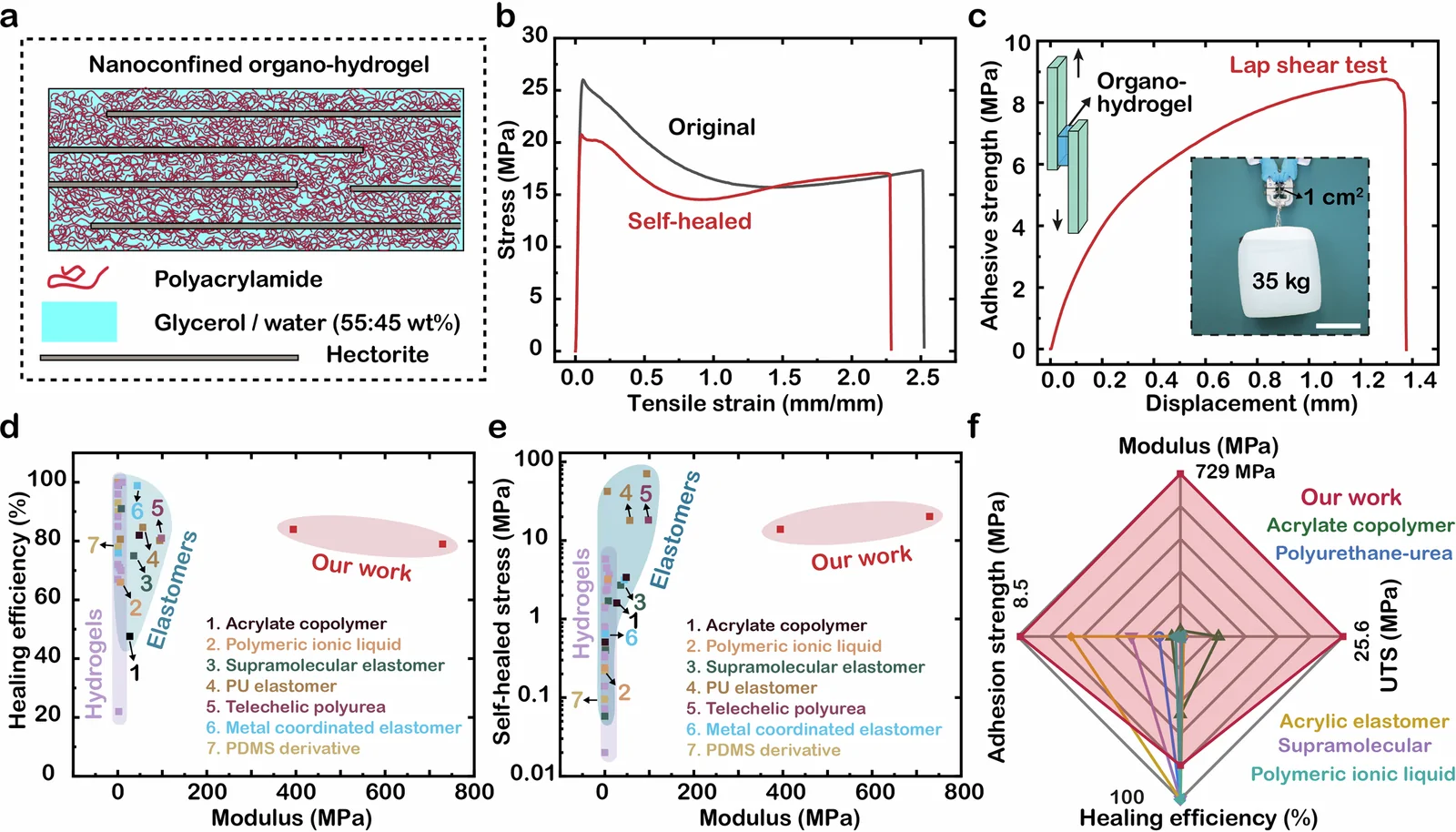
Application of nanoconfinement to elastomer-like organo-hydrogels. **a**, Schematic illustration of Hec-PAAm organo-hydrogels containing glycerol/water solvent mixture. **b**, Tensile stress-strain curves of pristine and self-healed Hec-PAAm organo-hydrogels. Self-healing in side-by-side geometry for 24 hours at 60 °C with overlapped distance of 2 mm. **c**, Lap-shear adhesion tests of organo-hydrogels on glass. Inset shows photograph of a piece of 1 cm^2^ organo-hydrogel holding a weight of 35 kg in lap shear configuration. Scale bar: 20 cm. **d**, Self-healing of nanoconfined organo-hydrogels outperforms self-healing elastomeric systems in terms of Young’s modulus. **e**, Ultimate tensile stress of self-healed organo-hydrogels outperforms reported self-healing elastomeric systems and hydrogels in terms of Young’s modulus. **f**, Radar map showing superior Young’s modulus, UTS, self-healing efficiency and adhesion strength on glass in the nanoconfined organo-hydrogel compared to reported self-healing elastomeric adhesives. Results in d-f are averaged from 3 measurements. All organo-hydrogels contained 2.7 wt% Hec and 67 wt% PAAm in a glycerol/water (55:45) solvent mixture.

The stiffening effect can also be achieved in other types of hydrogel, such as polydimethylacrylamide (Supplementary Fig. 28), where the modulus can be increased fourfold by the addition of 1.5 wt% hectorite. Furthermore, the nanoconfinement effect can be utilized to design organohydrogel systems (Extended Data Fig. 7 and Supplementary Figs. 29–32). The organohydrogel containing 2.7 wt% hectorite and 67 wt% PAAm exhibits outstanding mechanical and adhesive properties compared with self-healing elastomers in the literature (Extended Data Fig. 7d–f and Supplementary Tables 5 and 6). For instance, the organohydrogel possesses a Young’s modulus of 729 MPa, UTS of 25.6 MPa, efficient self-healing and high adhesion strength of up to 8.5 MPa. Although the Hec-PAAm hydrogel swells strongly in water (Supplementary Fig. 33), the applicability of the nanoconfined strategy in a wet or dry environment can be extended by various approaches, such as ionic crosslinking (Supplementary Fig. 34), or by forming organohydrogels (Supplementary Fig. 35).

**Extended Data Fig. 8:**
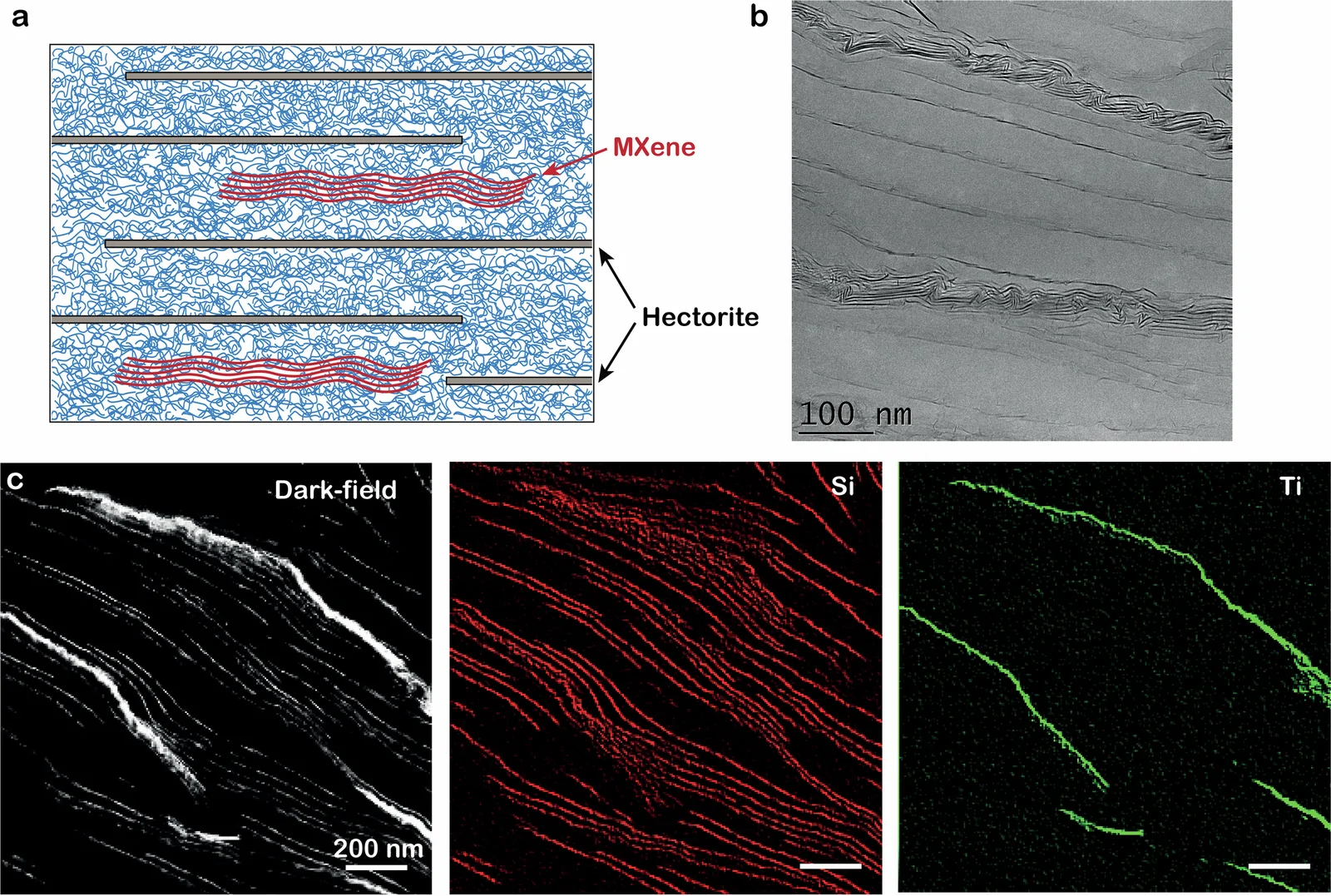
TEM characterization of Hec-MX-PAAm hydrogel. **a**, Schematic illustration of MXene distribution among hectorite. **b**, Cryo-TEM image of Hec-MX-PAAm hydrogel containing 1.5 wt% Hec, 1.5 wt% MXene, and 62 wt% PAAm. **c**, Dark field TEM image of the hydrogel cross-section with corresponding element mappings.

Additionally, the nanoconfined hydrogel also allows the incorporation of other types of nanomaterial, for example, MXene (MX) nanosheets^55^, between the hectorite nanosheets to achieve synergistic effects (Fig. 5d). For example, 1.5 wt% MXenes with a diameter of around 1 μm were mixed with 1.5 wt% hectorite to form the nanoconfined Hec-MX-PAAm hydrogel. The MXene nanosheets are uniformly distributed inside the nanoconfined hydrogel as characterized by TEM and energy-dispersive X-ray spectroscopy (EDX) (Extended Data Fig. 8). As a result, the high strength and stiffness of the hydrogel are preserved in the MXene-doped hydrogel (Fig. 5d), where the *E* value of the hydrogel is 16 MPa, whereas the UTS value is 4.3 MPa. It is noted that PAAm polymerized only in the presence of MXene without the hectorite scaffold did not form a mechanically robust hydrogel (Fig. 5d). Due to the presence of the infrared (IR)-reflective MXene^55^, the Hec-MX-PAAm hydrogel shows thermal camouflage capability, where the apparent surface temperature measured by an IR camera differs by up to 16 °C from the real temperature of the bulk of the hydrogel at 60 °C (Fig. 5e). The hydrogel also possesses unconventional electromagnetic interference (EMI)-shielding properties in the gigahertz range (Fig. 5f). At 5.76 GHz, for instance, the total shielding effectiveness (SE_T_) increased from 1.9 dB to 9.3 dB and the absorption component (SE_A_) increased from 1.4 dB to 5.0 dB by adding 1.5 wt% MXene to the nanoconfined hydrogel with a thickness of only 0.5 mm. To sum up, the nanoconfinement strategy can be applied to other types of gel and allows further functionalities to be incorporated, which will open up new avenues in the engineering of soft materials.

## Conclusion

We have demonstrated a general strategy to fabricate strong, stiff and self-healing hydrogels based on highly entangled polymers in the monodomain of co-planar nanoconfinement. The confinement is imposed between hectorite nanosheets, which spontaneously form nematic liquid crystallinity (that is, co-planar alignment) on one-dimensional dissolution. The high-AR hectorite ensures controllable uniform interlayer spacings around 100 nm, which can be further shear oriented into a macroscopic unidirectional monodomain. Once the confinement approaches the dimension of the highly entangled PAAm chains, a dramatic increase in the Young modulus of the hydrogel up to 50 MPa is observed, which is one order of magnitude higher than a non-confined hydrogel, whereas the UTS value reaches up to 4.2 MPa. Despite the high modulus, the hydrogels possess excellent self-healing properties, with 33% recovery of UTS in the end-to-end geometry and almost 100% in the side-by-side geometry. In particular, the hydrogel also shows strong binding to various substrates such as glass and metals, showing an adhesive strength of up to 0.49 MPa. The unique properties of the nanoconfined hydrogel allow the robust assembly of complex three-dimensional shapes, showing potential for additive manufacturing. In addition, the nanoconfinement effect can be extended to other types of monomer and solvent, such as organohydrogels with outstanding mechanical and adhesive properties. The incorporation of functionalities is demonstrated by an MXene-doped nanoconfined Hec-PAAm hydrogel with thermal camouflage and EMI-shielding capabilities. The nanoconfinement strategy, thus, allows high stiffness in a self-healing hydrogel comparable to biological tissues like skins and opens new avenues to engineer soft-matter properties and to design complex shapes, relevant for applications like artificial skin and soft robotics.

## Methods

### Materials

Acrylamide (≥99%), 2-hydroxy-4′-(2-hydroxyethoxy)-2-methylpropiophenone (Irgacure 2959, 98%), *N*,*N*-dimethylacrylamide (99%), ammonium persulfate (≥98%), sodium chloride (≥99%), silicon dioxide (fine granular), lithium fluoride (powder, 300 mesh), magnesium fluoride (99.9%), sodium fluoride (99.99%), hydrochloric acid (ACS Reagent, 37%), 3-aminopropyltriethoxysilane (APTES, 99%), 3-(trimethoxysilyl)propyl acrylate (≥92%), toluene (≥99.5%), silicon oil (CAS no. 63148-62-9, viscosity 5 cSt), methacrylic acid (for synthesis) and glycerol (≥99%) were purchased from Sigma-Aldrich. Iron(iii) chloride (anhydrous, 98%) was purchased from Thermo Scientific. Magnesium oxide (99.95%) was purchased from Alfa Aesar. Fluorescein-PEG-acrylate (cat. no. FL044009-2K) was purchased from Biopharma PEG. Toluene was dehydrated by 4 Å molecular sieves for 48 h before use. Deionized water (18.2 MΩ; Millipore Direct-Q 3 UV) was used in all the experiments. Laponite RD was provided by BYK Additives & Instruments. Ti_3_AlC_2_ MAX powder (particle size, <40 µm) was purchased from Carbon-Ukraine.

### Synthesis of hectorite

The synthetic clay sodium fluorohectorite (Hec) was synthesized with high-purity reagents of silicon dioxide, lithium fluoride, magnesium fluoride, magnesium oxide and sodium fluoride in a gas-tight molybdenum crucible via melt synthesis according to a published procedure^38^. To improve the homogeneity of the charge density of the clay nanosheets and the uniformity of the related intracrystalline reactivity, the melt synthesis was followed by long-term annealing at 1,045 °C for 6 weeks^56^. The material featured a cation exchange capacity of 1.27 mmol g^−1^.

### Preparation of small hectorite

For the fabrication of the small hectorite platelets, a 0.5 wt% suspension of hectorite was sonicated for 15 min in an ice bath with an ultrasonic device UIP1000hd (Hielscher Ultrasonic), equipped with an ultrasonic horn BS2d22 and a booster B2-1.2, at 20 kHz with a maximum output power of 1,000 W.

### Preparation of Mt dispersion

Mt was dispersed in water (0.5 wt%) and stirred vigorously overnight, then left for 4–5 days to allow the sedimentation of large agglomerates and finally decanted. The homogeneous supernatant containing delaminated Mt nanosheets was centrifuged at 25,000*g* for 30 min to yield a concentrated dispersion of 5 wt%, which was used as the stock solution to prepare the hydrogels.

### Synthesis of Ti_3_C_2_T_*x*_ MXene

Ti_3_C_2_T_*x*_ MXene nanosheets were prepared using our reported method modified from the minimally intensive layer delamination procedure^57^. First, 2 g of lithium fluoride dispersed in 5 ml of deionized water was added to 30 ml of 10 M hydrochloric acid by stirring at 35 °C. A suspension of 2 g Ti_3_AlC_2_ MAX powder in 5 ml deionized water was added dropwise to the above solution. After 24 h, the resulting sediment was washed with water until its pH reached 6. The obtained slurry was then exfoliated by 1 h vortexing in 35 ml water. Well-dispersed Ti_3_C_2_T_*x*_ MXene nanosheets in water were obtained by centrifugation at 2,360*g* for 15 min. After determining the concentration using the gravimetric method, the supernatant was then concentrated to 8 wt% by centrifugation at 10,864*g* and redispersed in water to achieve the desired concentrations.

### Preparation of nanoconfined hydrogels

The typical precursor solution of the nanoconfined hydrogel containing 62 wt% PAAm and 1.5 wt% hectorite was prepared by dissolving 1.3 g of acrylamide and 5 mg of Irgacure 2959 photoinitiator in 0.8 g of 4 wt% hectorite dispersion. The hectorite dispersions were prepared by dissolving a certain amount of hectorite in water by stirring for 2 days. For other concentrations of hectorite, higher or lower concentrations of hectorite dispersions were used to prepare the precursor solution. Afterwards, the precursor solution was degassed for 3 min by nitrogen bubbling to eliminate the oxygen inhibition of radical polymerization. The degassed solution was then transferred into a nitrogen-protected glovebox, followed by centrifugation for 10 s to remove the bubbles. The glovebox provided an inert atmosphere during the flow orientation and polymerization process to prevent oxygen back-diffusion into the precursor solution, which would inhibit the radical polymerization. Then, the precursor solution was slowly injected into a mould with 0.5 mm thickness at a flow rate of around 5.2 mm s^−1^ to provide macroscopically uniform orientation of the LC domains. The mould consisted of parafilm spacers between two parallel substrates, one made of glass (Corning) and the other one of polystyrene (VWR, Petri Dish, PS). The plastic substrate was used to ensure the intact demoulding of the hydrogel after synthesis, as removing a hydrogel synthesized between two glass substrates causes damage to the hydrogel due to high adhesion. Both substrates were treated with argon plasma (Pico, Diener Electronic) for 10 min before use, to ensure hydrophilicity and facilitate the flow of the precursor solution. The hydrogels were polymerized under UV illumination (365 nm, 6 W, Spectroline E-Series) in the glovebox for 1 h. The temperature of the sample during polymerization was controlled at 18 °C on a Peltier stage (Digital Heating Cooling Drybath, Thermo Scientific) to prevent overheating of the hydrogel during the exothermic polymerization process. For the MXene-containing hydrogels, 20 µl ammonium persulfate stock solution (0.2 g ml^−1^) was added as the initiator to the mixture of acrylamide, hectorite and MXene. The MXene-containing hydrogel was polymerized at 60 °C for 2 h, where the mould was sealed in a plastic bag to prevent water evaporation.

### Preparation of organohydrogels

For the organohydrogel, the hectorite was first dispersed in a glycerol/water (55/45 w/w) mixture, which was then used to prepare the precursor solution containing 2.4 mg g^−1^ Irgacure 2959; certain concentration of hectorite; and 62 wt%, 65 wt% or 67 wt% of acrylamide. The precursor solution was flow oriented in the same way as the Hec-PAAm hydrogel. The polymerization was carried out under UV irradiation for 1 h at 50 °C to prevent the precipitation of monomers, where the mould was sealed in a plastic bag to prevent water evaporation.

### Preparation of Fe^3+^-coordinated hydrogel

For the preparation of Fe^3+^-coordinated hydrogel, 1.3 g acrylamide in the original Hec-PAAm recipe was replaced by 1.04 g acrylamide and 0.26 g methacrylic acid (MAAc). The synthesis protocol was the same as that in the Hec-PAAm hydrogel. Thus, the final hydrogel contained 1.5 wt% hectorite, 49.6 wt% PAAm and 12.4 wt% polymethacrylic acid. The as-prepared hydrogels were immersed in 5 wt% FeCl_3_ solution for 1 day, followed by washing with Milli-Q water for another 2 days. Afterwards, the hydrogels were dried under ambient conditions for 1 day before being rehydrated in water.

### Silanization of glass slides

The glass slides were cleaned with deionized water and then treated with O_2_ plasma (Pico, Diener Electronic) for 10 min. For APTES modification, the glass slides were immediately immersed in a 10% (v/v) APTES solution in anhydrous toluene for 24 h at room temperature. Finally, the modified glass slides were rinsed with anhydrous toluene to remove unreacted APTES and dried in a nitrogen flow. For acrylate modification, the slides were stored overnight in an evacuated desiccator containing 100 µl of 3-(trimethoxysilyl)propyl acrylate at 1 × 10^−1^ mbar. Subsequently, the liquid silane was removed, and the desiccator was further evacuated to 1 × 10^−3^ mbar for 2 h to remove any unbound silane on the glass surface. The silanized glass slides were stored in a sealed vial in the fridge before use.

### Mechanical tests

The mechanical properties of the hydrogels were measured using an Instron 5567 universal tensile tester. The stretching speed was 1,000% min^−1^ using a 100 N load cell. The mechanical properties of the Hec-PAAm organohydrogels were measured using an Instron 4204 at 1,000% min^−1^ with a 100 N load cell. Rectangular-shaped samples were cut from the as-prepared hydrogel films with a thickness of 0.5 mm. The width and length of the hydrogels were measured using a digital caliper before tests. The two ends of the hydrogel samples were glued between two plastic sheets using super glue (Loctite, Henkel). Subsequently, the hydrogels were coated with silicon oil before the measurement to reduce the evaporation of water. Young’s modulus was calculated by the slope of the linear area of the tensile test curve. All the measurements were carried out under 30% relative humidity at 25 °C. Each measurement was repeated five times. The creep test was performed at a step stress of 1 MPa, followed by maintaining the tensile stress for 4 h at 50% relative humidity. The creep strain rate was extracted from the creep strain data, where the data after 500 s were smoothed using a Savitzky–Golay filter with 20 points of window in OriginPro 2023b to reduce the noise level^58^.

### Lap-shear adhesion test

The lap-shear measurements of Hec-PAAm hydrogels were performed using Instron 5567 at a speed of 5 mm min^−1^. The lap-shear measurements of Hec-PAAm organohydrogels were performed using Instron 4204 at a speed of 5 mm min^−1^. For the adhesion test, two pieces of glass slides (Corning) were rinsed with deionized water and treated for 10 min under argon plasma. The hydrogel with a size of 1 cm × 1 cm and a thickness of 0.5 mm was prepared by injecting the precursor solution into the reaction cell consisting of the two pretreated glass slides and a parafilm spacer, followed by UV polymerization for 1 h at 18 °C. The organohydrogels were prepared with a size of 0.5 cm × 1 cm and a thickness of 0.5 mm. The organohydrogels were stored at 80% relative humidity for 24 h before measurement. Glass substrates with 2 mm thickness (ultrawhite glass, Luoyang Guluo Glass) have been used for the lap-shear test of organohydrogels.

### Self-healing of nanoconfined hydrogels

The hydrogel was cut into two pieces with a razor blade and attached to each other in the end-to-end or side-by-side manner (overlapped length, 1 or 2 mm) after a minor amount of water (3 µl) was applied to the overlapped area. Afterwards, the attached hydrogels were kept in sealed glass vials at room temperature to prevent the dehydration of hydrogels. For the side-by-side self-healing, the stress is calculated based on the original film thickness (0.5 mm). The self-healing of organohydrogels was performed in the same way.

### Calculation of water content

The water content was measured from the weight loss before and after drying the samples at 80 °C under a vacuum for 3 days. The measurement was repeated three times.

### SEM

The SEM images of the hydrogels were recorded on a Sigma VP instrument (ZEISS) at 3 kV. The hydrogels were frozen in liquid nitrogen, cut and then freeze dried for 24 h. The dried samples were coated with 4 nm Au–Pd before the SEM measurements. The hectorites were characterized by a ZEISS Ultra Plus device at an operating voltage of 3 kV. Samples were prepared by drop coating a 0.001 wt% solution on a plasma-treated silicon wafer. The hectorite nanosheets were sputtered with 10 nm carbon. ImageJ (version 1.54g) was used to analyse the area of the nanosheets, and at least 50 nanosheets were evaluated.

### Cryo-TEM

The cross-section slices of the hydrogel were prepared by freezing the hydrogel in liquid propane and then cutting using a Leica UltraCut 7 microtome equipped with a Diatome Cryo35 diamond knife at –120 °C with a thickness of 100 nm. The cryo-TEM images were captured using a JEOL JEM-3200 FSC device operating at an acceleration voltage of 300 kV and a temperature of –187 °C. ImageJ (version 1.53u) was used to analyse the separation of the nanosheet, and at least 50 data points were evaluated.

### Scanning TEM–EDX

The scanning TEM–EDX measurement was conducted using a JEM-2800 (JEOL) device in the annular dark-field mode operated at an acceleration voltage of 200 kV.

### SAXS

Small-angle X-ray measurements were conducted using a Xenocs Xeuss 3.0 SAXS/WAXS system (Xenocs SAS) that includes a microfocus X-ray source (sealed tube) operating at 50 kV and 0.6 mA with a Cu target and a multilayer mirror that yields a parallel beam with a nominal wavelength of 1.542 Å (combined Cu Kα1 and Cu Kα2 characteristic radiation). The beam was collimated by a set of variable slits and the beam size at the sample was 0.4 mm during the experiment. The absence of a beam stop enables the direct measurement of sample transmission. Background scattering from the sample holder was normalized and subtracted from the data according to sample transmission. The data were acquired using an area detector (Eiger2 R 1M, Dectris) in the evacuated chamber. The sample-to-detector distance was calibrated by measuring the diffraction from a known LaB_6_ standard sample. The distance between the detector and the capillary is 1.1 m. The azimuthal profiles were obtained using a *q* range between 0.009 Å^−1^ and 0.1 Å^−1^ for all the samples. The numerical values were obtained from a Gaussian function that was fitted to the data. The data were smoothed using the two-point fast Fourier transform method in OriginPro 2023b. The calculation of the orientation factor (*f*) is provided in Supplementary Methods.

### Static light scattering

The particle size distribution of the hectorite was measured by laser diffraction according to the ISO 13320 standard using Retsch LA-950 (Horiba).

### AFM

The samples were prepared by the slow evaporation of a few drops of a diluted suspension (0.02 g l^−1^) on a Si wafer under ambient conditions. The surface topography has been determined by atomic force microscopy (AFM) measurements. The images were acquired using a Dimension Icon (Bruker Nano) in the PeakForce Tapping mode in air. The AFM images were processed using NanoScope Analysis 1.80 (Bruker Nano). The topography was flattened by subtracting a first-order polynominal background using a threshold to exclude the platelets from flattening. Nanosheet heights were determined by means of a ‘step tool’ in the NanoScope Analysis (version 1.8) software.

### SEC

The size-exclusion chromatography (SEC) measurements were performed on an Agilent 1260 Infinity II Multi-Detector GPC/SEC system, which contains light scattering (two measurement angles of 15° and 90°) and refractive-index detectors. Separation was performed on two serially coupled Agilent 7.5 mm × 300 mm PL aquagel-OH MIXED-M columns with a 7.5 mm × 50 mm PL aquagel-OH guard column. Here 0.1 M NaNO_3_ was used as the eluent at a flow rate of 0.7 ml min^–1^, and the injection volume was 100 µl. The temperature of the column and detector was kept at 30 °C. The relative molar mass was obtained by the calibration of the columns using narrow-dispersity polystyrene sulfonate standards with molar masses ranging from 208,000 to 3,300,000. The samples were prepared by dissolving hydrogels in water to form a 1.5 mg ml^–1^ solution by stirring at 50 °C for 3 days. To remove the clay, the samples were filtered through a 5 µm syringe filter once and through a 0.45 µm syringe filter twice before the measurements.

### FRAP

To study the dynamics of PAAm chains in the nanoconfined hydrogels, FRAP was performed using a Nikon Ti-E inverted microscope controlled with µManager (v. 2.0). The hydrogel samples were prepared by adding 0.1 wt% fluorescein-PEG-acrylate relative to the acrylamide monomer in the precursor solution containing 1.5 wt% hectorite and 62 wt% PAAm. The hydrogel was illuminated using 470 nm light from a laser diode illuminator (89 North) at 2% power level with 100 ms exposure time. The emission light was collected between 485 nm and 535 nm using a ×20/0.75-numerical-aperture objective lens and ×1 tube lens with a Hamamatsu ORCA Flash4.0LT camera. For photobleaching, the Gataca Systems iLas 2 unit coupled to a 100 mW OBIS LX 405 nm laser was used. Specifically, four spots were bleached with 50% laser power level for 1 s per spot. The diameter of a spot was approximately 5 µm. After photobleaching, the fluorescence recovery data were collected at a 10 s interval for about 4 h. The free fluorescein-PEG-acrylate in the acrylamide monomer solution was also measured with 0.2 s bleaching time and 50 ms capture interval. The free fluorescein-PEG-acrylate sample was prepared by sandwiching 2 µl of the solution between two glass cover-slips. Data from the bleached spots and the non-bleached control region were analysed using Fiji (v. 2.3.0) software, and the acquisition photobleaching correction was performed using the ratio between the bleached area and the control region in OriginPro (v. 10.0.5.157, Academic). The data were normalized, and the half-time of recovery was manually extracted from the recovery curve. The calculation of the apparent diffusion coefficient is provided in Supplementary Methods.

### Thermal imaging of hydrogels

The hydrogels were equilibrated on a hotplate set at different temperatures for 1 min and then measured by an infrared camera (FLIR T420BX, close-up lens with 50 µm resolution).

### EMI-shielding measurement

EMI-shielding capacity measurements were performed in the frequency range between 5.3 GHz and 8.17 GHz by a coaxial air-line method using a microwave network analyser (Aglient E8363A). The rectangular samples with a size of 30 mm × 20 mm were used for EMI-shielding tests. Scattering parameters (S_11_ and S_21_) were obtained to calculate the EMI-shielding effectiveness. The detailed calculations of SE_T_ and its reflection and absorption components are provided in Supplementary Methods.

## Online content

Any methods, additional references, Nature Portfolio reporting summaries, source data, extended data, supplementary information, acknowledgements, peer review information; details of author contributions and competing interests; and statements of data and code availability are available at https://doi.org/10.1038/s41563-025-02146-5.

## Supplementary information


Supplementary InformationSupplementary Figs. 1–35, Tables 1–6, Methods, discussion and refs. 1–63.


## Source data


Source Data Fig. 1Source data for Fig. 1c–e.
Source Data Fig. 2Source data for Fig. 2a–f.
Source Data Fig. 3Source data for Fig. 3b–f.
Source Data Fig. 4Source data for Fig. 4b–d,f.
Source Data Fig. 5Source data for Fig. 5d–f.


## Data Availability

The datasets generated during and/or analysed during the current study are available from the corresponding authors upon request. Source data are provided with this paper.
