# Supplementary material for: Stiff and self-healing hydrogels by polymer entanglements in co-planar nanoconfinement
Source: Nat Mater. 2025 Mar 7;24(4):599–606. doi: 10.1038/s41563-025-02146-5 (PMC11961364; doi:10.1038/s41563-025-02146-5)
Supplement: Supplementary file 1 — Supplementary Figs. 1–35, Tables 1–6, Methods, discussion and refs. 1–63. [file 41563_2025_2146_MOESM1_ESM.pdf]

nature materials

Supplementary information

https://doi.org/10.1038/s41563-025-02146-5

Stiff and self-healing hydrogels by polymer entanglements in co-planar nanoconfinement

In the format provided by the authors and unedited

Table of Contents

1. Supplementary Methods 2

2. Supplementary Discussions... 4

3. Supplementary Tables... 8

3. Supplementary Figures 14

5. Supplementary References... 49

1. Supplementary Methods

Calculation of Herman's orientation factor (*f*)

The Herman's orientation factor (*f*) was calculated from the obtained function using the given equation:

$$f = \frac{1}{2}\left(\frac{3\int_0^{\frac{\pi}{2}} I(\phi)cos^2\phi sin\phi d\phi}{\int_0^{\frac{\pi}{2}} I(\phi)sin\phi d\phi} - 1\right),$$

where the $I(\phi)$ is the intensity at an azimuthal angle of ϕ.

Calculation of apparent diffusion coefficient

The apparent diffusion coefficient D_{app} was calculated according to Axelrod et al.[1]

$$D_{\text{app}} = \frac{0.88\, r^2}{4\, \tau_{1/2}}$$

where r is the radius of the bleached spot. The average D_{app} was 0.0004 ± 0.000008 μm^2 s^{-1} for the Hec-PAAm hydrogel and 0.001 ± 0.0006 μm^2 s^{-1} for the PAAm hydrogel (n = 4). For the free fluorophore, the average D_{app} was 5.7 ± 2.1 μm^2 s^{-1}. The data are presented as mean values ± standard deviations from four measurements.

Calculation of radius of gyration and countor length

The radius of gyration[2] can be calculated according to the following equation:

$$\langle R_g^2 \rangle^{1/2} = 7.49 \times 10^{-2} M^{0.64} (\text{Å})$$

The contour length (L) can be calculated according to the following equation:

$$L = Nl$$

Where N is the number of monomers, and l is the length of monomer (0.3 nm)[3].

Calculation of hydrogel properties using Halpin Tsai's model.

The Young's modulus of the hydrogel was calculated using the Halpin Tsai model[4] shown below:

$$E_c = E_m\left[\frac{1 + \zeta\eta\,\phi_f}{1 - \eta\phi_f}\right]$$

Where E_c = composite modulus, E_m = modulus of unfilled matrix, and ϕ_f = filler volume fraction.

$$\eta = \frac{E_f/E_m - 1}{E_f/E_m + \zeta}$$

$$\zeta = 2(l/t)$$

where E_f is the filler modulus, for which hectorite is 142 GPa[5] and Laponite® and montmorillonite is 178 GPa[6,7]. l is characteristic length of the filler, t is the thickness, and the ratio of l/t thus presents the aspect ratio of clay.

Calculations of the total EMI SE (SE_T) and reflection (SE_R) and absorption (SE_A) components

The total EMI SE (SE_T) and its reflection (SE_R) and absorption (SE_A) components are calculated as follows[8]:

$$SE_T = SE_R + SE_A$$

$$SE_T = -10\log(|S_{21}|^2)$$

$$SE_R = -10\log(1 - |S_{11}|^2)$$

$$SE_A = -10\log(\frac{|S_{21}|^2}{1 - |S_{11}|^2})$$

2. Supplementary Discussions

Discussion on comparison of hectorite with conventional nanoplatelet or nanosheets: The hectorite in hydrogels is intrinsically liquid crystalline with well-defined separations between the nanosheets. This is in strong contrast to conventional hydrogels containing 2D nanoplatelets or sheets, which are either laterally too small (e.g., Laponite®, montmorillonite) to form well-defined and oriented, monodomains without applying strong magnetic or electric fields[9,10], or have too heterogeneous surface chemistry to allow ideal delamination and a uniform nanosheet spacing (e.g., MXene or graphene oxide). Besides, the shear-oriented hectorite dispersion shows drastically slower relaxation in contrast to small nanoplatelets with rapid randomization kinetics[11,12].

Discussion on water content of Hec-PAAm hydrogel: The as-prepared Hec-PAAm hydrogels have a water content of 27 wt% (Supplementary Fig. 2), determined by the dry weight. This is lower than the theoretical value of 36.5 wt% due to evaporation of water during the preparation process and residue water trapped in dried samples.

Discussion on difference of Hec-PAAm hydrogel from nacre-like materials: Compared to nanoconfined Hec-PAAm hydrogels, the nacre-like materials have vastly different periodicities (e.g., 1- 3 nm separation between tablets, or 10 μm separation), low polymer content (~ 5 wt%), glassy polymer in dry state, and low fracture strain at 2 – 3%.[13–15]

Discussion on previously reported nanocomposite system: Similar effect of mechanical reinforcement has been observed previously in dry polymer nanocomposites, though it should be noted that the reported nanoconfinement only existed in a much smaller scale around few nanometers on glassy polymer chains[15]. In addition, a high-modulus (43.2 MPa) nanocomposite hydrogel system consisting of PNIPAm and jammed Laponite® has been reported at a Laponite® concentration of 23.2 wt% by filtration methods[16]. However, a power-law dependence of the modulus with the Laponite® concentration was observed, indicating a distinct stiffening mechanism compared to the nanoconfined hydrogel based on LC nematic hectorite.

Discussion on Halpin-Tsai model on the aspect ratio of filler: The Halpin-Tsai model for composite material shows a dependence of modulus on the aspect ratio of filler material, but the resulted values deviate largely from experimental results for high-aspect ratio filler (Supplementary Fig. 6 and Supplementary Table 1).

Discussion on the influence of the strain rate and water content on the mechanical properties of the hydrogels: The influence of strain rates on the mechanical properties of Hec-PAAm hydrogels are shown in Supplementary Fig. 7. The hydrogels exhibit decreased moduli of 25 and 47 MPa at lower strain rates of 100 % min^{-1} and 500 % min^{-1}, respectively. Comparably, strain rates between 500 and 1000 % min^{-1} are most commonly used in reported stiff and self-healing hydrogels, as summarized in Supplementary Fig. 8. Besides, the water content also has a strong influence on the mechanical properties, in particular the Young's modulus as shown in Supplementary Fig. 9. Dehydrating the as-prepared Hec-PAAm hydrogel to a water content of 22.5 wt% leads to stiffening and increased modulus of 186 MPa, while hydration of the hydrogel to a water content of 33.8 wt% leads to softening and decreased modulus of 14 MPa. This is in accordance with previous report on chemically crosslinked PAAm hydrogels containing lamellar bilayers[17].

Discussion on FRAP measurement: The half time of recovery is 60 ± 1 min for Hec-PAAm hydrogel and 20 ± 11 min for pure PAAm hydrogel. This time scale is faster than the self-healing across broken surfaces due to the smaller probed area (bleached spot diameter of 5 μm). The apparent diffusion coefficient[18] of Hec-PAAm hydrogel calculated from FRAP measurement is 4×10^{-4} $\mu m^2 s^{-1}$, much lower than reported values (0.8 – 4 $\mu m^2 s^{-1}$) in polymer coacervates[18], thus reflecting the slower chain diffusion dynamics of the highly entangled PAAm chains in the nanoconfinement.

Discussion on comparison to conventional self-healing hydrogels: Self-healing of only surface cracks or under significant re-processing (e.g., by melting of crystalline domains) is not included in Fig. 3e&f, such as hydrogel microfibers fabricated by pultrusion spinning[19] or hydrogels with high crystallinity[20]. The highest reported modulus of self-healing hdyrogels shown in Fig. 3e&f is around 10 MPa in a π-π supramolecular hydrogel[21], which, however, requires heating and softening to achieve 100% self-healing (Fig. 3e). Besides, some self-healing hydrogels, despite having a low water content, e.g., at 12 wt%[22], are much softer with a modulus of close to 1 MPa compared to Hec-PAAm.

Discussion on control experiments on adhesion test: The negative surface charge on glass surface did not seem to play a role in the enhanced adhesion, as the hydrogel has also shown strong adhesion on (3-aminopropyl)triethoxysilane modified positively charged glass surface, reaching

an adhesive strength of 0.34 MPa (Fig. 4d). Furthermore, the addition of free sodium ions (NaCl) to the pure PAAm gel showed no observable effects on the adhesion strength (0.15 MPa) compared to pristine PAAm hydrogel (Fig. 4d), which proves that the sodium introduced as the counterions of hectorite did not participate in enhancing the interfacial strength.

Discussion on comparison to self-healig and adhesive hydrogels: A polydopamine nanocomposite hydrogel has been reported with efficient self-healing and high shear adhesion strength, however, the material is rather weak with low UTS (0.3 MPa) and modulus (1.4 MPa)[23].

Discussion on anti-drying and anti-swelling capabilities: The pristine Hec-PAAm hydrogel swells significantly in water but does not dissolve at room temperature (Supplementary Fig. 33). By introducing ionic crosslinks via Fe^{3+} coordination between acrylic acid groups[24], the swelling stability can be significantly improved at the expense of self-healing properties, as shown in Supplementary Fig. 34. After equilibration in water for 2 days, the Fe^{3+} coordinated hydrogel showed a linear swelling of 20% and maintained a high modulus of 17.9 MPa and an UTS of 8.3 MPa. Additionally, the organo-hydrogel possess excellent anti-drying properties shown by the minor weight fluctuations over a period of 30 days as the relative humidity changed between 46 and 73%, with its mechanical properties fully preserved after storage (Supplementary Fig. 35). The results demonstrated above substantially expands the applicability of the nanoconfinement concept to soft systems requiring long-term exposure to air, such as soft robotics or artificial skins[25]. We also foresee the possibility to encapsulate the nanoconfined hydrogel with elastomeric coatings[26,27], which will endow further resistance to swelling or drying without changing the mechanical properties of the pristine hydrogel.

Disucssion on MXene-PAAm hydrogel: The AAm polymerized only in the presence of MXene without the hectorite scaffold did not form a mechanically robust hydrogel, but only a soft and viscous pasteas, as shown in Fig. 5d. This can be attributed to the incomplete delamination of the MXene nanosheets, which consequently fails to form an oriented monodomain LC state. The hectorite liquid crystallinity is preserved in the Hec-MX-PAAm hydrogel without aggregation, as both types of nanosheets are negatively charged.

Disucssion on EMI properties: The results shown in Fig. 5f serve as a proof of principle, and we expect further increase of the EMI capability by simply increasing the MXene concentration and/or

the thickness of the hydrogel, as demonstrated in a polyvinylalchohol/MXene hydrogel with much lower stiffness and UTS[28].

3. Supplementary Tables

Supplementary Table 1. Young's moduli calculated from Halpin-Tsai model for composite hydrogels prepared with nanoplatelets of different aspect ratios.

	Aspect ratio	Volume fraction* (%)	Halpin-Tsai Modulus (MPa)	Experimental modulus (MPa)
**Laponite**	20	0.656	7.0	5.2
**Mt**	150	0.656	16.3	6.5
**Small Hec**	440	0.656	36.4	11.7
**Hec**	22500	0.656	598.4	50.0

*Volume fraction equivalent to 1.5 wt% weight fraction.

Supplementary Table 2. Molecular weight of PAAm in different nanoconfined hydrogels.

	$\bar{M}_n$ (×10^6)	$\bar{M}_w$ (×10^6)	PDI
**Pristine PAAm**	0.93	1.8	1.9
**Hec (1.5 wt%)-PAAm (40 wt%)**	0.75	1.04	1.4
**Hec (1.5 wt%)-PAAm (50 wt%)**	0.99	1.83	1.8
**Hec (0.38 wt%)-PAAm (62 wt%)**	1.16	2.23	1.9
**Hec (0.76 wt%)-PAAm (62 wt%)**	1.01	2.0	2
**Hec (1.5 wt%)-PAAm (62 wt%)**	1.0	1.64	1.6
**Hec (3 wt%)-PAAm (62 wt%)**	1.1	1.91	1.7
**Hec (4.6 wt%)-PAAm (62 wt%)**	0.97	1.66	1.7

PDI: Polydispersity Index

Supplementary Table 3. Reference lists of self-healing hydrogels.

	Modulus (MPa)	Strain rate (%/min)	Stress (MPa)	Self-healing efficiency (%)
**Our work**	50	1000	2.94	100
**Our work**	47	500		
**Our work**	25	100		
**Double network PBMA- b -PMAA- b -PBM**[29]	2.2	840	10.5	22
**Polyelectrolyte PMPTC/PNaSS**[30]	5.4	833	3.7	66#
**π/ π stacking poly(MAA-co-OEGMA)**[21]	10	500	3.7	100
**PNIPAM/GNP**[31]	0.015*	1667	1.02	96#
**Dynamic bond poly(MAA-co-OEGMA)/EDA**[32]	7.2	833	4.3	70*
**PAAm/Ag@BACA**[33]	0.221	/	2.9*	85#
**Polyampholyte P(NaSS-*co*-DMAEA-Q)**[34]	0.1	833	0.14	99
**Hydrophobic domain PVA-CBA0.12**[35]	1.6	500	5.8	100
**Laponite/PDMAA**[9]	0.02*	333	0.14*	100
**PAAc/MXene**[36]	0.04*	/	0.5	88.3#
**Hydrogen bond PVA/PAAc**[37]	0.06*	417	0.1*	72
**Supramolecular hydrogel**[38]	0.12*	/	0.03	67*
**Ionic bonds/hydroboic association PPSB-HA/SDS**[39]	0.023*	250	0.354	100
**Metal-coordination P(AAm-*co*-AAc)/Fe^{3+}**[24]	4*	833	6.3	71*

#: self-healing efficiency was calculated according to strain. *: estimated value from plot.

Supplementary Table 4. Reference lists of self-healing and adhesive hydrogels.

	Modulus (MPa)	Stress (MPa)	Healing efficiency (%)	Adhesion strength (MPa)
**Our work****	51	4.2	86	0.49
**Polydopamine nanocomposite**[23]	1.4*	0.3	97	0.61
**Zwitterionic nanocomposite**[40]	0.05*	0.09	80	0.094
**Tannic acid-based hydrogel**[41]	0.01*	0.054*	61	0.053
**Cellulose nanocomposite**[42]	0.04	0.102	92	0.065*

*: estimated value from plot. ** Sample containing 4.6 wt% Hec.

Supplementary Table 5. Reference lists of self-healing elastomer.

	Modulus (MPa)	Stress (MPa)	Self-healing efficiency (%)
**Our work**	729	25.6	79
**Our work**	394	16.6	84
Fe-Hpdca-PDMS[43]	0.54	0.23	90#
PDMS-$MPU_{0.4}$-$IU_{0.6}$[44]	0.62	1.7*	78#
Polyurethane-GO[45]	93.9	88	80.2
TA-WS2/PU[46]	5.7	52.3	80.6
Hydrogen-bonding brush polymer[47]	35.7	3.77	75#
$Zn(Hbimcp)_2$-PDMS[48]	43.68	3.22	98.9#
$Zn(OTf)_2$-PDMS[49]	1.1	0.65*	76#
PIL-Zn elastomer[50]	6.67	4.85*	66*
PUDS[51]	56.05	21.7	84.7
Telechelic polyurea[52]	97.9	22.5	81
PDMS–SS–IP–BNB[53]	0.034	0.095*	93#
PBMA-PEA[54]	48	3.8*	82#
Poly(TA-DIB-Fe)[55] Supramolecular elastomer	7.78	1.87	91*
SHSME[56] Supramolecular elastomer	1.2	0.4*	99#
U-PDMS-Es[57]	0.037	0.227	100#

#: self-healing efficiency was calculated according to strain. *: estimated value from plot.

Supplementary Table 6. Reference lists of self-healing and adhesive hydrogels.

	Modulus (MPa)	Stress (MPa)	Healing efficiency (%)	Adhesion strength (MPa)
**Our work**	729	25.6	79	8.5
**Polyurethane elastomer**[58]	1.21	0.41	100	1.13
**P(PEA-co-IBA)**[59] **Acrylate copolymer**	27	6*	47.6	0.46*
**Poly(TA-DIB-Fe)**[60] **Supramolecular elastomer**	0.081	0.058*	100	2.6*
**P(AAc-co-BA)**[61] **Acrylic elastomer**	0.59	0.51	100	5.8*
**PIL/TFSI**[62] **Polymeric ionic liquid**	0.071	0.24	100	0.25

*: estimated value from plot.

3. Supplementary Figures

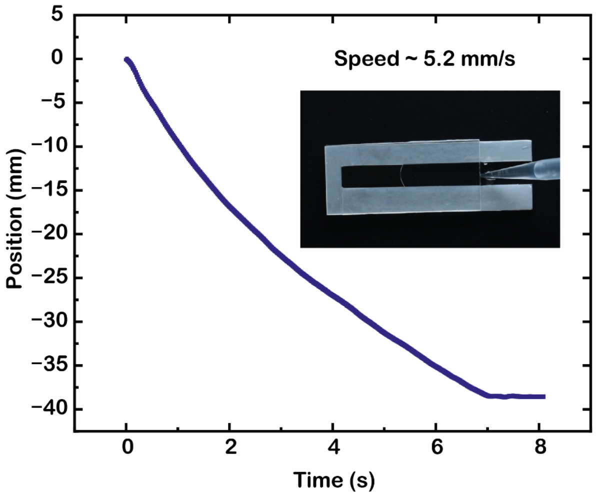

Supplementary Figure 1. The trajectory of injected precursor solution frontline along the flow direction. Inset shows the photo of the precursor solution being injected. Mold thickness: 0.5 mm.

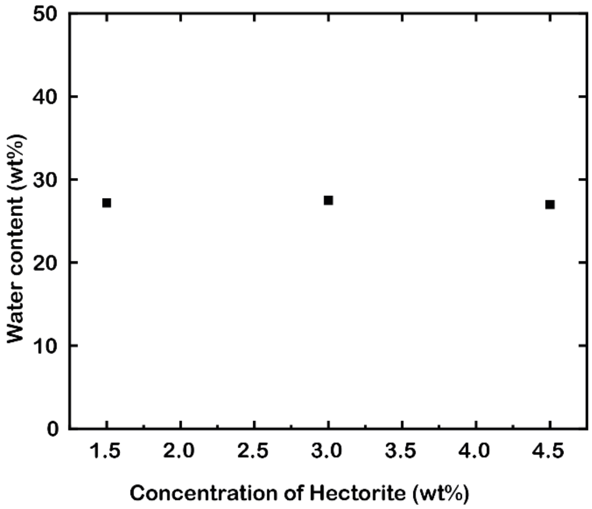

Supplementary Figure 2. Water contents of Hec-PAAm hydrogels at different hectorite concentrations. Data are presented as mean values ± standard deviations from three samples dried at 80°C in a vacuum oven (<0.1 mbar) for 3 days to constant weight.

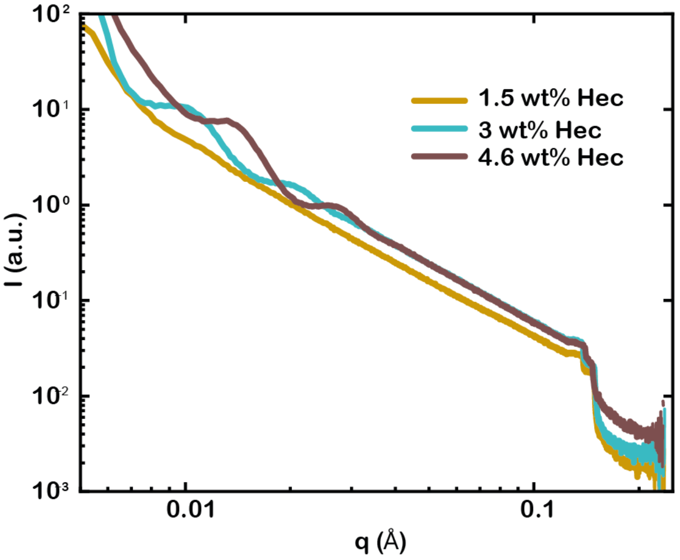

Supplementary Figure 3. SAXS profiles of nanoconfined Hec-PAAm hydrogels containing different concentrations of hectorites. The PAAm concentration was 62 wt%.

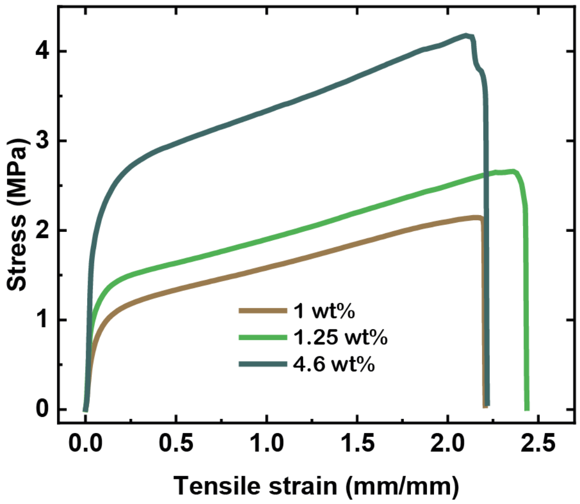

Supplementary Figure 4. Tensile stress-strain curve of nanoconfined Hec-PAAm hydrogels containing different concentrations of hectorite.

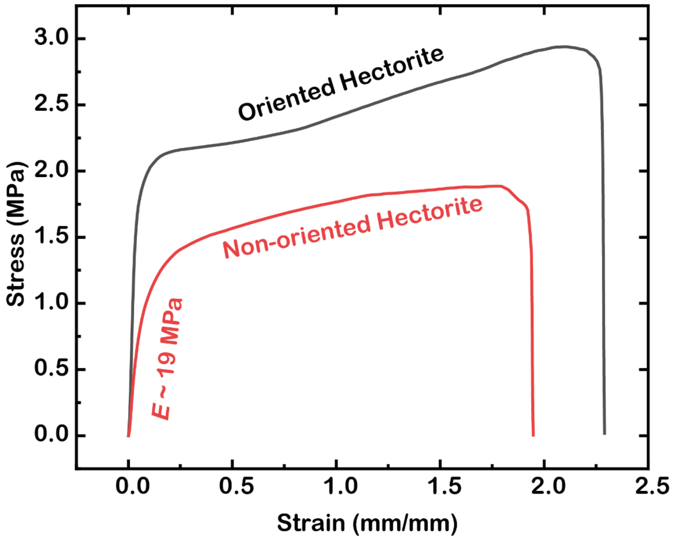

Supplementary Figure 5. Tensile tests of Hec-PAAm containing oriented or non-oriented hectorite.

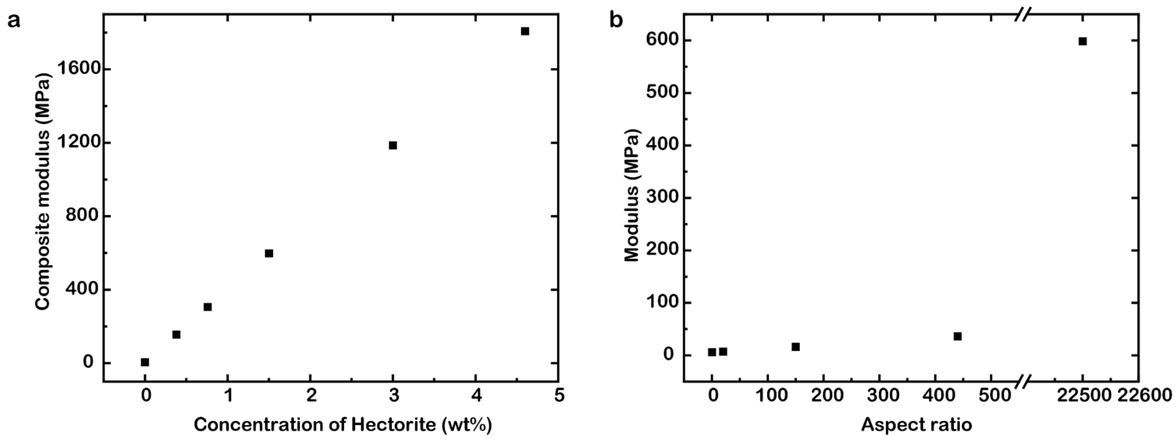

Supplementary Figure 6. Calculated Young's moduli based on Halpin-Tsai equation. (a) Calculated Young's moduli with the weight fraction of the hectorite. (b) Calculated Young's moduli with different types of clay.

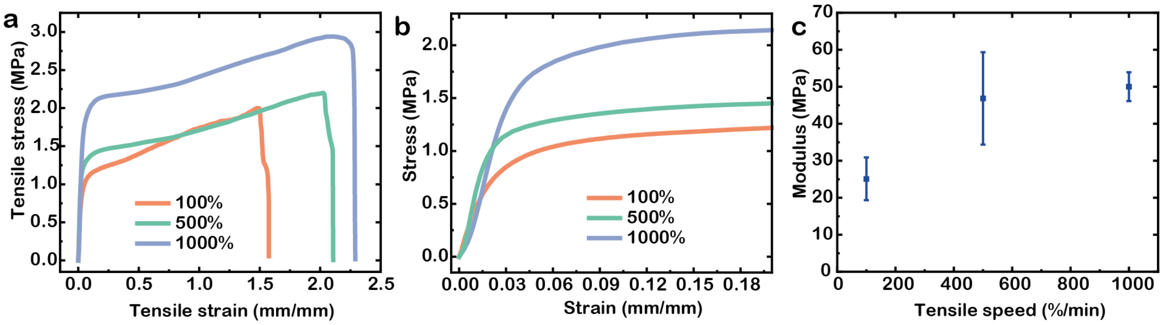

Supplementary Figure 7. Tensile tests of Hec-PAAm hydrogels under different strain rates. (a) Stress-strain curves. (b) Zoom-in of the low-strain region. (c) Young's moduli with mean values ± standard deviations from 5 samples. The hydrogel contained 62 wt% PAAm and 1.5 wt% hectorite.

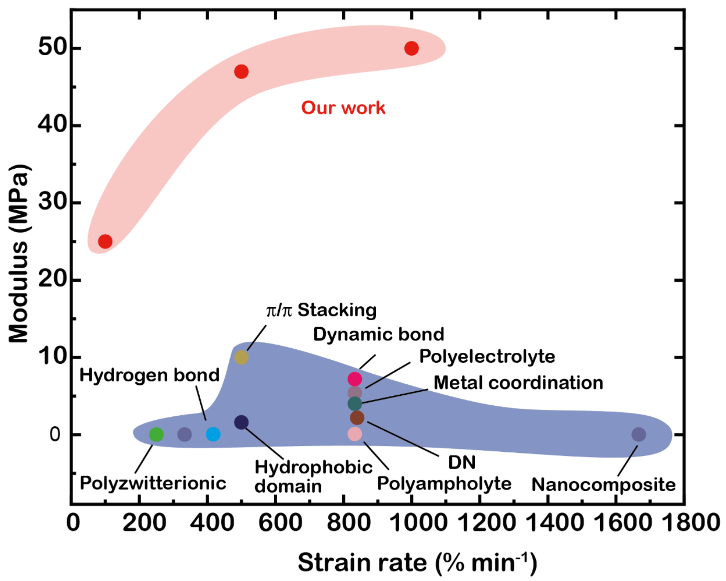

Supplementary Figure 8. Summary of Young's moduli and corresponding strain rates in reported stiff and self-healing hydrogels.

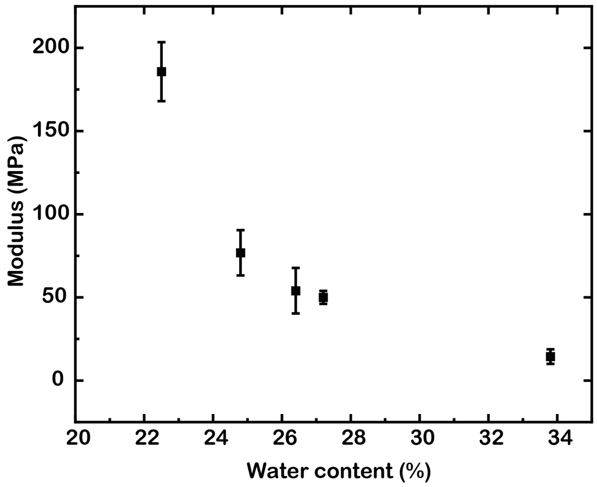

Supplementary Figure 9. The relationship of the Young's moduli with water content. The as-prepared Hec-PAAm hydrogels containing 1.5 wt% hectorite and 62 wt% PAAm were hydrated or dried to reach different water contents. As-prepared water content: 27 wt%. Data are presented as mean values ± standard deviations from 3 samples.

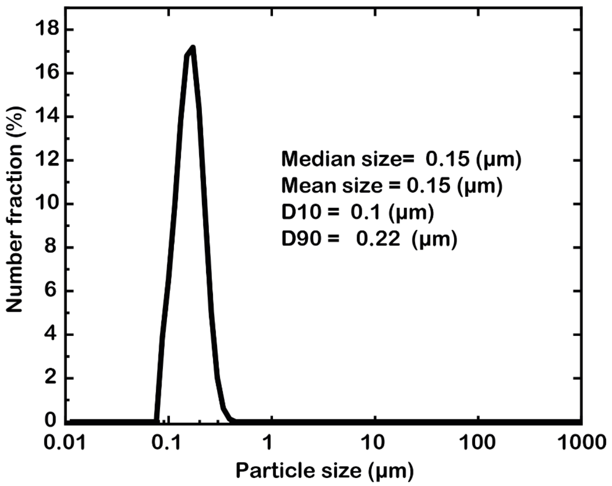

Supplementary Figure 10. SLS Characterization of Mt.

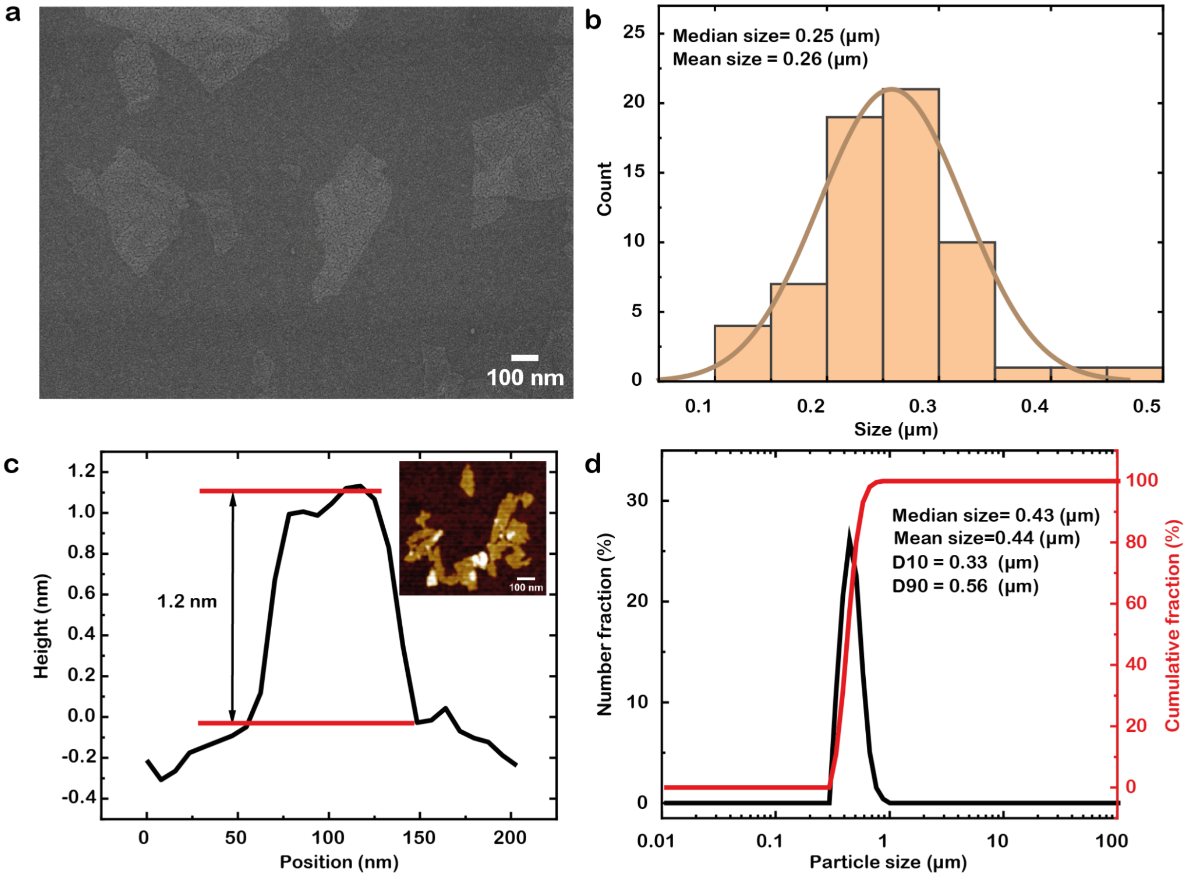

Supplementary Figure 11. Characterization of small hectorite. (a) SEM image of small hectorite nanosheets and their corresponding size distribution (b). Data are presented from 64 measurements. (c) AFM image of hectorite nanosheets with a height of about 1.2 nm (including sodium cation). (d) SLS measurement of the size distribution of small hectorite dispersion.

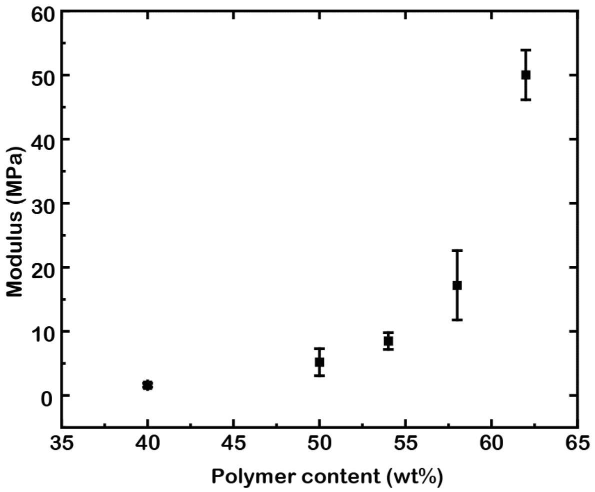

Supplementary Figure 12. Young's moduli of nanoconfined Hec-PAAm hydrogels prepared with different polymer contents. Hectorite concentration: 1.5 wt%. Data are presented as mean values ± standard deviations from 5 samples.

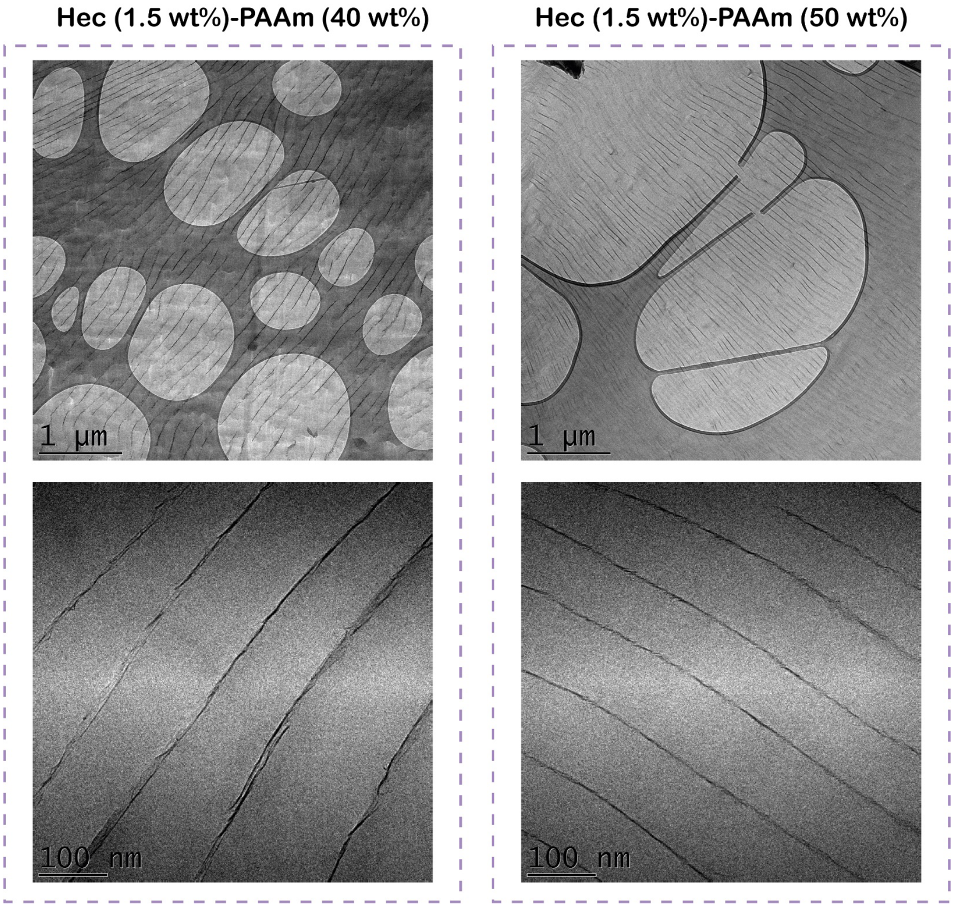

Supplementary Figure 13. Cryo-TEM images of nanoconfined Hec-PAAm hydrogels containing 40 wt% and 50 wt% PAAm, and 1.5 wt% hectorite.

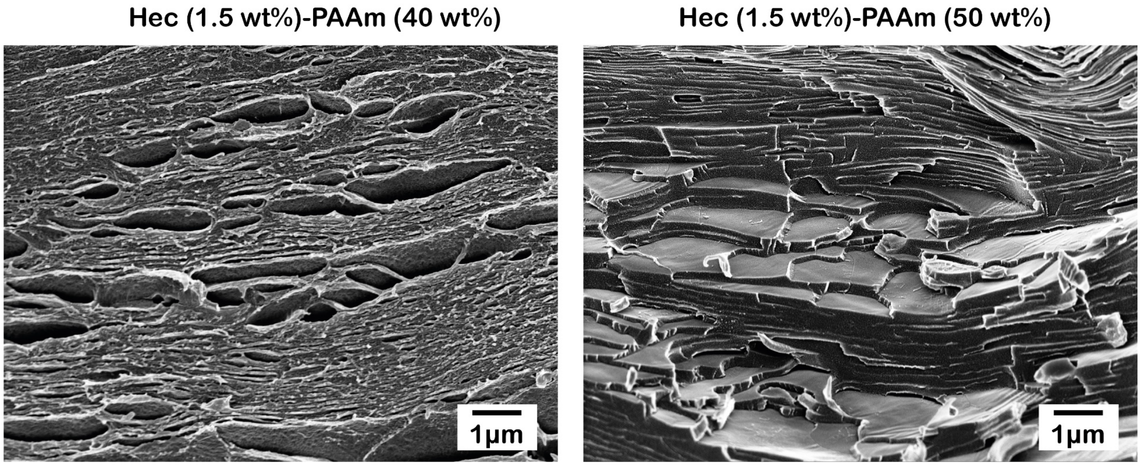

Supplementary Figure 14. Cross-sectional SEM images of Hec-PAAm hydrogels containing different polymer content.

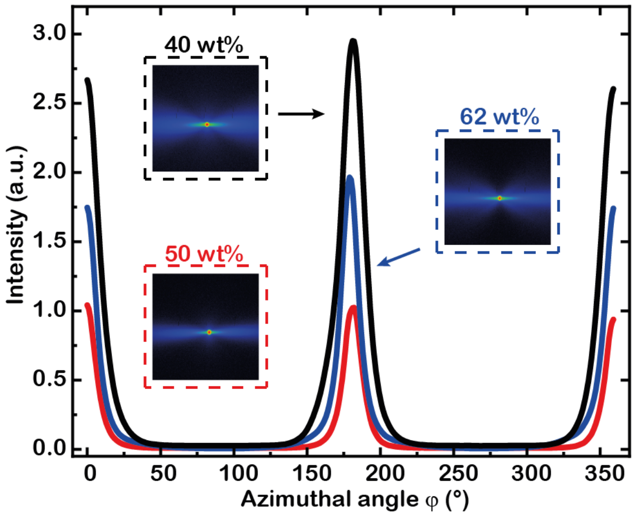

Supplementary Figure 15. Azimuthal plots and 2D SAXS patterns of Hec-PAAm hydrogels containing different polymer contents. Hectorite concentration: 1.5 wt%.

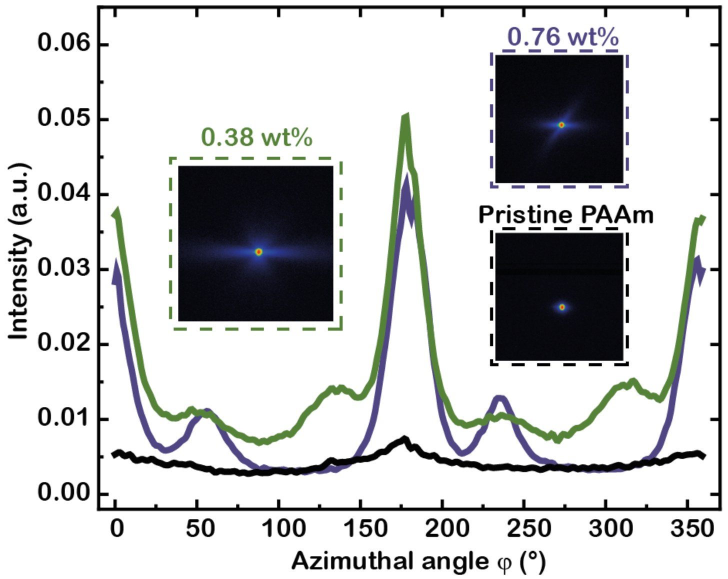

Supplementary Figure 16. Azimuthal plot and 2D SAXS patterns of pristine 62 wt% PAAm hydrogel and Hec-PAAm hydrogels containing 0.38 wt% and 0.76 wt% hectorite.

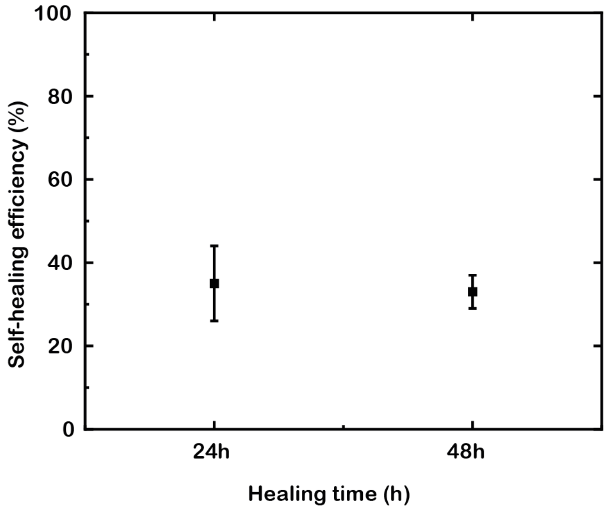

Supplementary Figure 17. Self-healing efficiency of the end-to-end self-healed hydrogels under different healing time. Data are presented as mean values ± standard deviations from 5 samples.

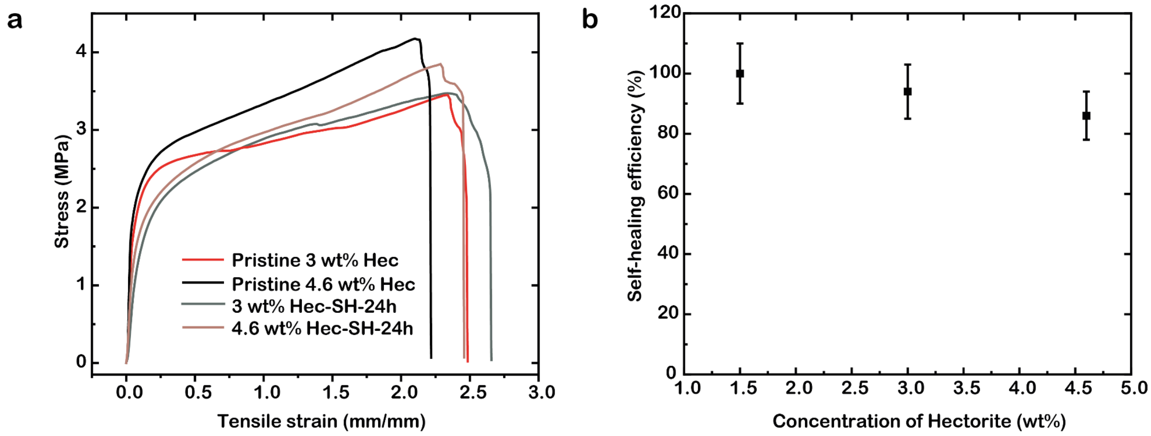

Supplementary Figure 18. Self-healing of Hec-PAAm hydrogel containing 3 wt% / 4.6 wt% Hec and 62 wt% PAAm with an overlapped length of 2 mm. (a) Tensile stress-strain curve of pristine and self-healed Hec-PAAm hydrogel side by side after 24h. (b) Self-healing efficiency of the corresponding self-healed Hec-PAAm hydrogel. Data are presented as mean values ± standard deviations from 5 measurements. Self-healing efficiencies higher than 100% are presented as 100%.

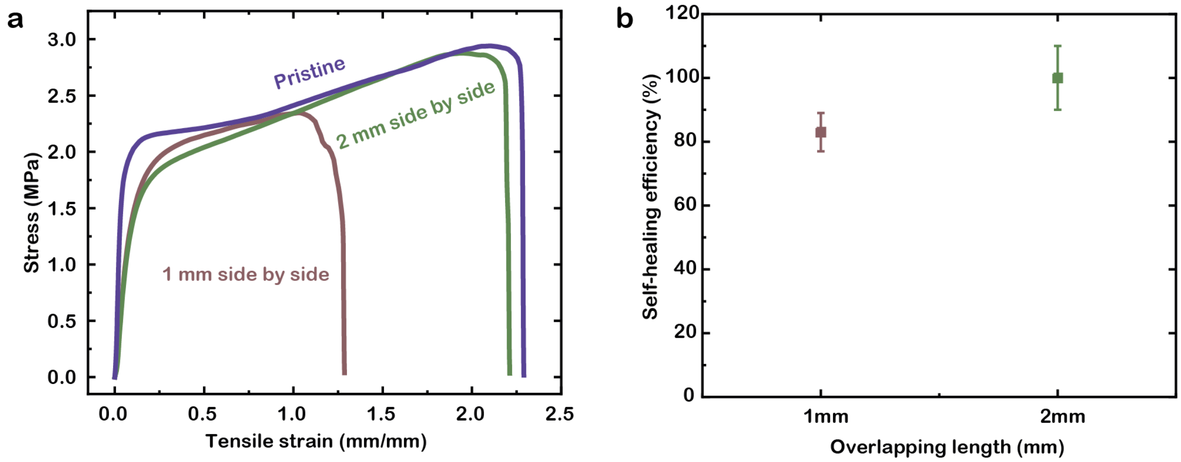

Supplementary Figure 19. Self-healing of Hec-PAAm hydrogel containing 1.5 wt% Hec and 62 wt% PAAm with different overlapped lengths. (a)Tensile stress-strain curve of original and side-by-side self-healed samples (24 h) with different overlapped length (1 mm and 2 mm). (b) Self-healing efficiency of the corresponding hydrogels. Data are presented as mean values ± standard deviations from 5 measurements. Self-healing efficiencies higher than 100% are presented as 100%.

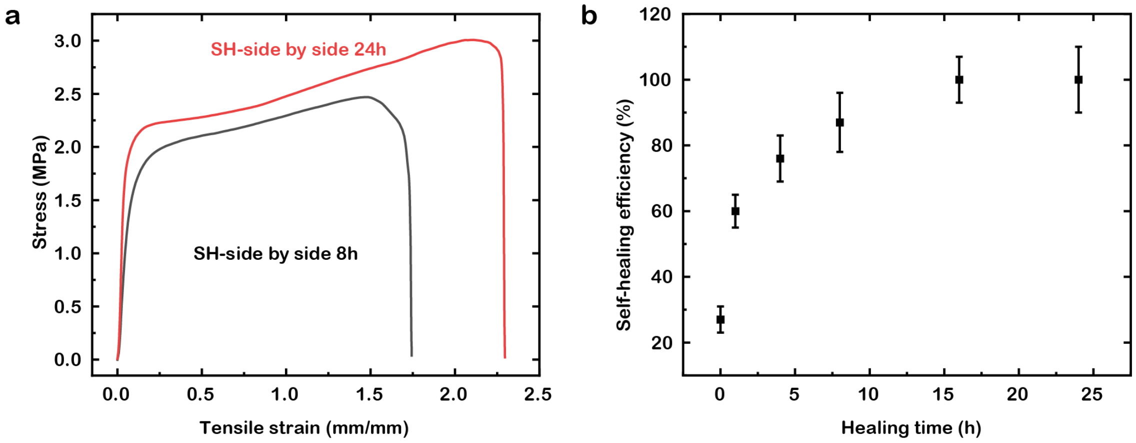

Supplementary Figure 20. Kinetics of side-by-side self-healing. (a) Tensile stress-strain curve of side-by-side self-healed hydrogels under different healing times. (b) Self-healing efficiency of the side-by-side self-healed hydrogels under different healing time. Data are presented as mean values ± standard deviations from 5 measurements. The hydrogels contained 1.5 wt% hectorite and 62 wt% PAAm. Overlapped length: 2 mm. Self-healing efficiencies higher than 100% are presented as 100%.

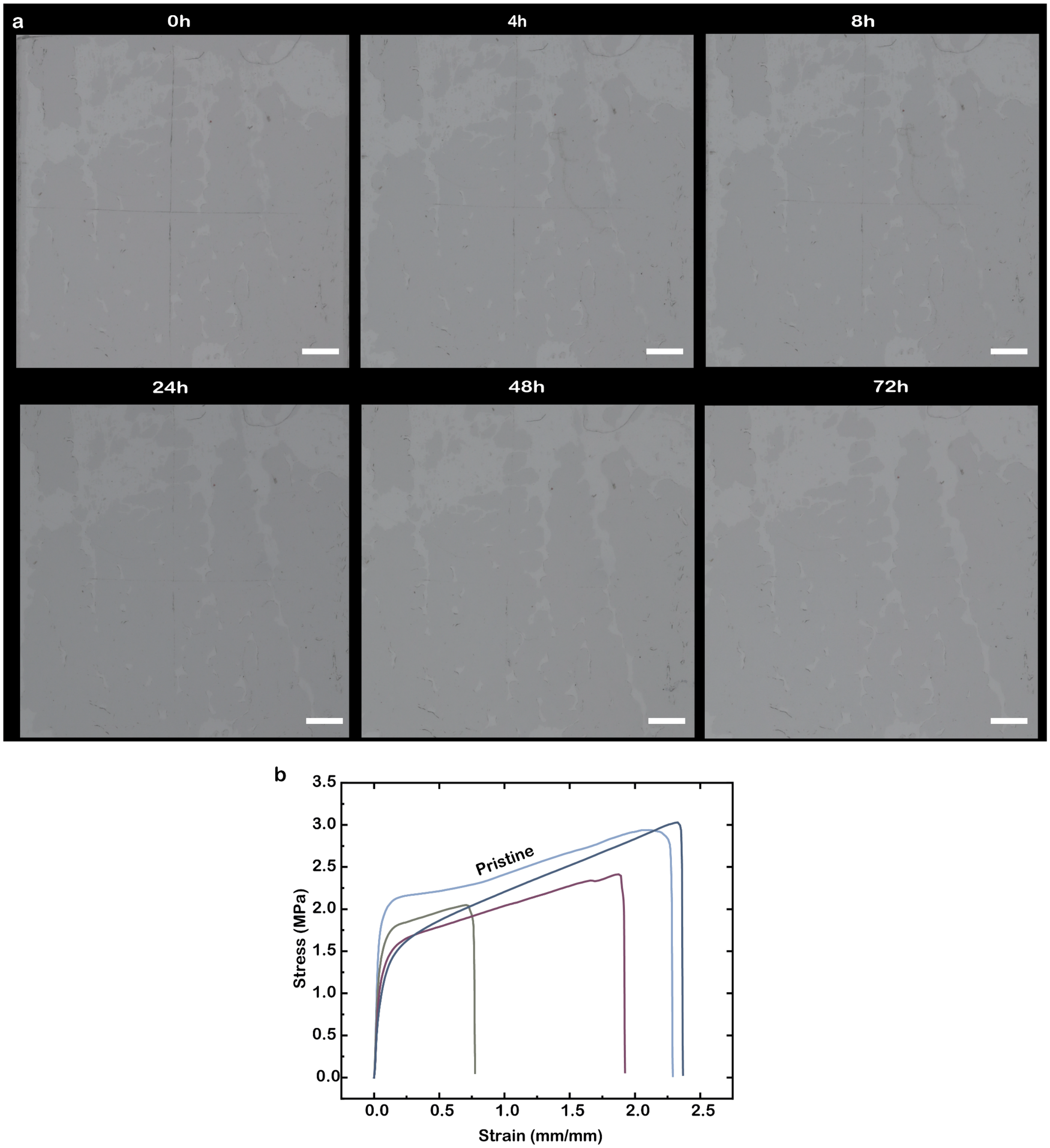

Supplementary Figure 21. Self-healing of the scratches on the surface of the Hec (1.5 wt%)-PAAm (62 wt%) hydrogel. (a) Photographs of the self-healing process of surface scratches in cross shape. Cut depth: 40 μm. (b) Tensile curves of surface scratched samples with time, where the crack was perpendicular to the stretching direction. Scale bars: 1 mm.

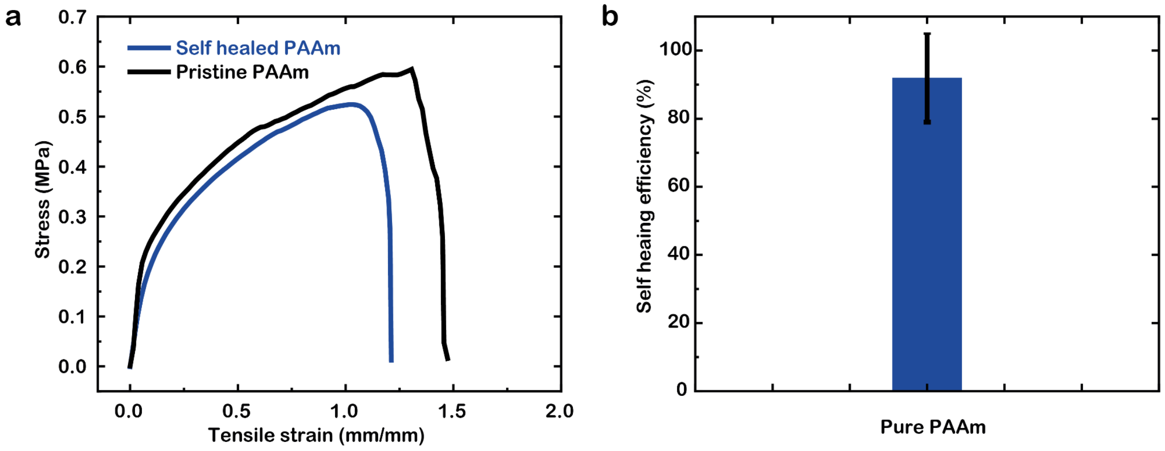

Supplementary Figure 22. Self-healing of pristine PAAm hydrogel with an overlapped length of 2 mm. (a) Tensile stress-strain curve of pristine and self-healed 62 wt% PAAm hydrogels after 24h. (b) Self-healing efficiency of the corresponding self-healed 62 wt% PAAm hydrogel. Data are presented as mean values ± standard deviations from 5 measurements.

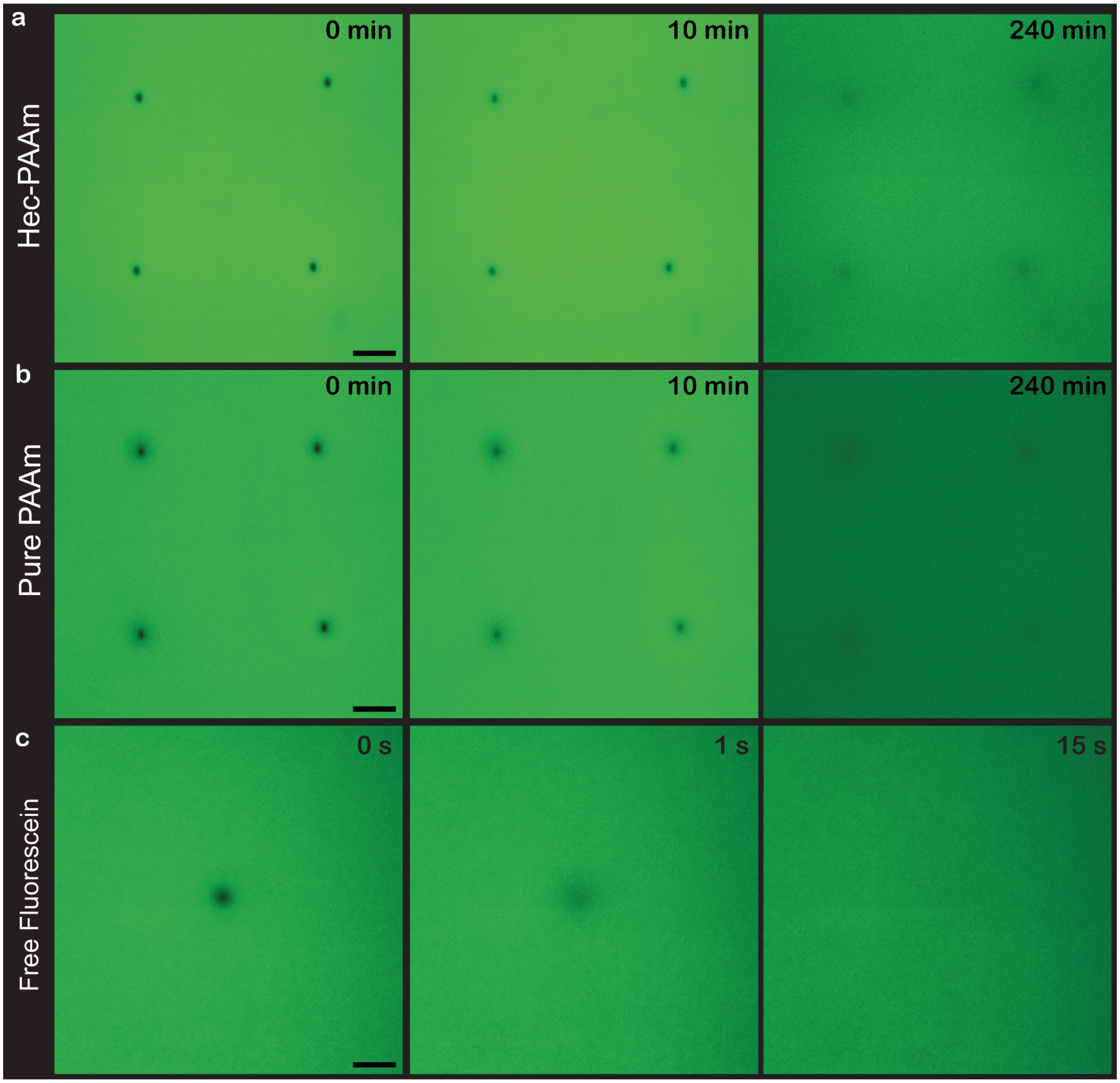

Supplementary Figure 23. Representative fluorescence images of the FRAP measurement. (a) Fluorescein-labeled Hec (1.5 wt%)-PAAm (62 wt%) hydrogel. (b) Fluorescein-labeled pure PAAm (62 wt%) hydrogels. (c) Free Fluorescein-PEG-Acrylate in 62 wt% AAm monomer solution. Scale bars: 50 μm.

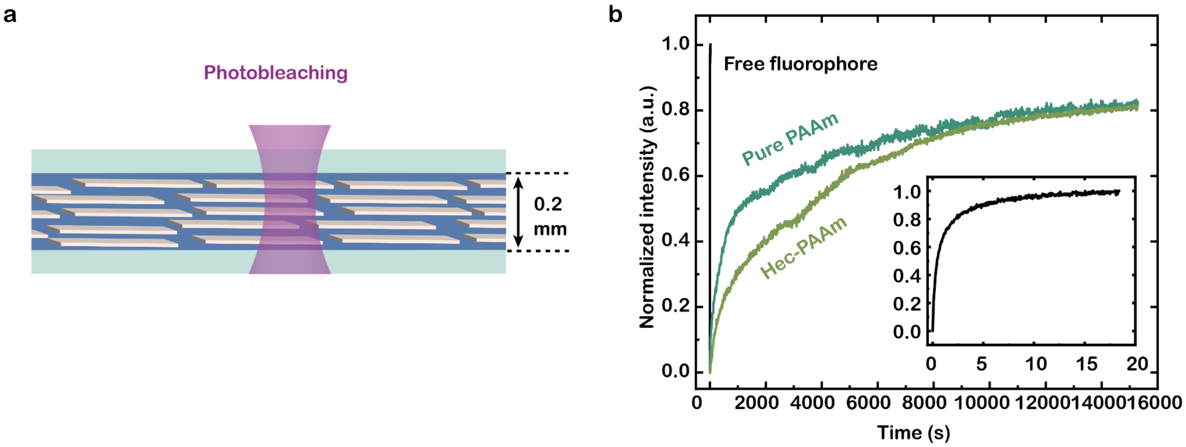

Supplementary Figure 24. Fluorescence intensity recovery after photo-bleaching. (a) Illustration of the photobleaching process, (b) Recovery of fluorescence in fluorescein-labeled Hec (1.5 wt%)-PAAm (62 wt%) hydrogel, pure PAAm hydrogel, and free Fluorescein-PEG-Acrylate dissolved in 62 wt% AAm solution. Inset shows zoom-in of the data acquired from free Fluorescein-PEG-Acrylate solution.

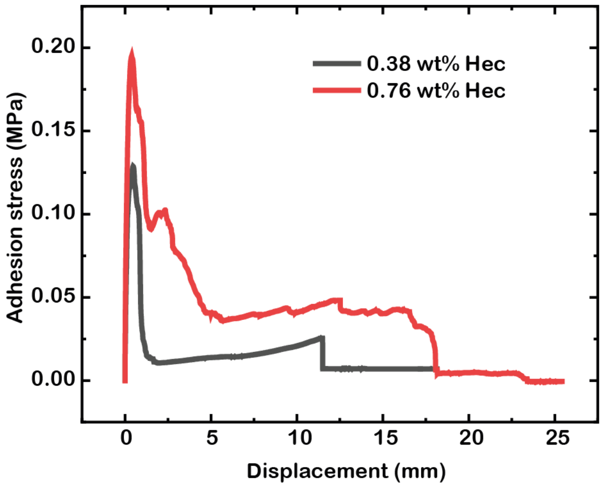

Supplementary Figure 25. Lap-shear adhesion stress of 0.38 wt% and 0.76 wt% Hec-62wt% PAAm.

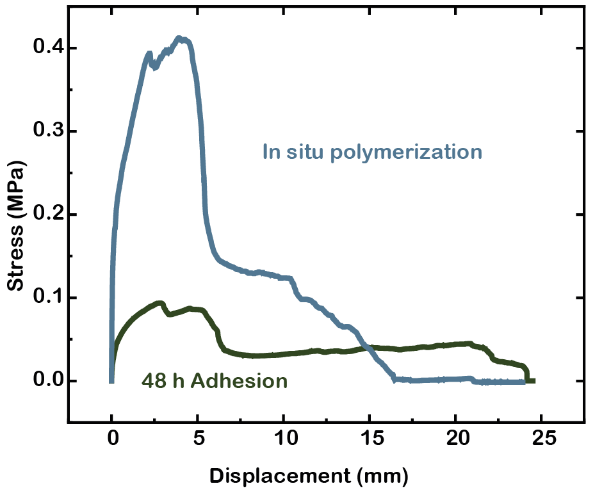

Supplementary Figure 26. Adhesion measurements of *in situ* formed nanoconfined hydrogel and nanoconfined hydrogels post-adhered for 48 hours between glass substrates. The hydrogel contained 3.0 wt% hectorite and 62 wt% PAAm.

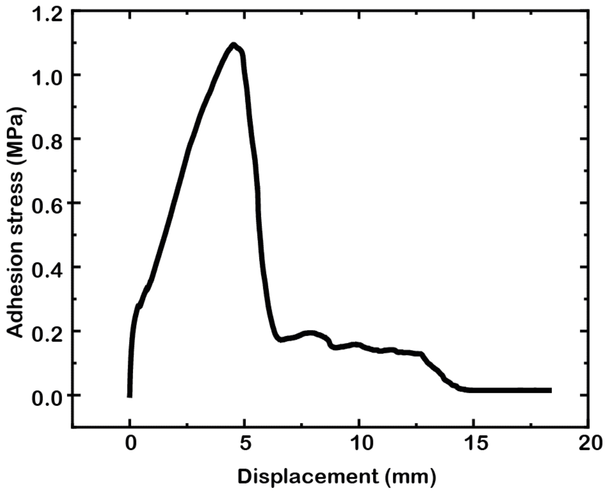

Supplementary Figure 27. Lap-shear adhesion stress of nanoconfined Hec-PAAm hydrogel on acrylate-silane modified glass substrates. The hydrogel contained 3.0 wt% hectorite and 62 wt% PAAm.

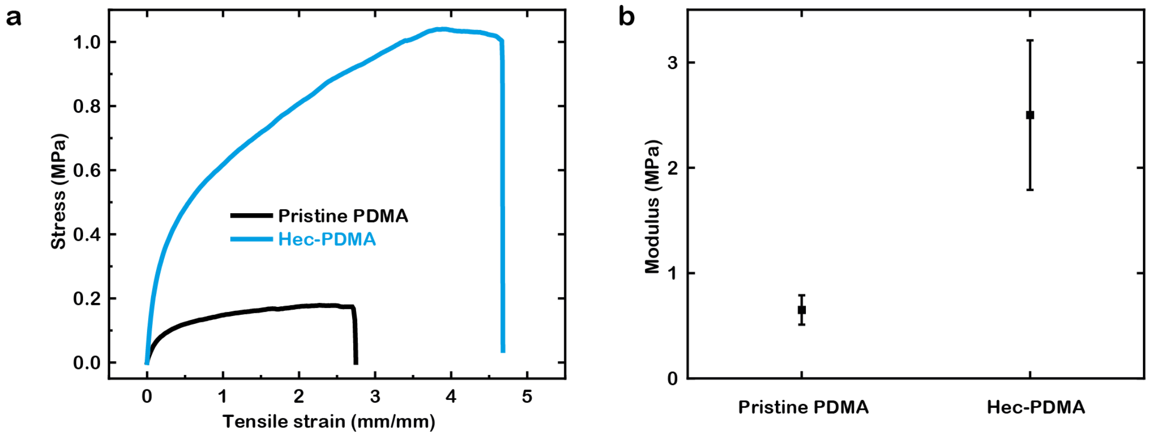

Supplementary Figure 28. Nanoconfinement enhances the elastic modulus of Hec-PDMA hydrogel containing 1.5 wt% hectorite and 62 wt% polydimethylacrylamide (PDMA) compared to pristine PDMA hdyrogel. (a) Tensile stress-strain curves of PDMA hdyrogels. (b) Young's moduli of nanoconfined PDMA hydrogels compared to pristine PDMA hydrogel. The moduli are presented as mean values ± standard deviations from 5 samples.

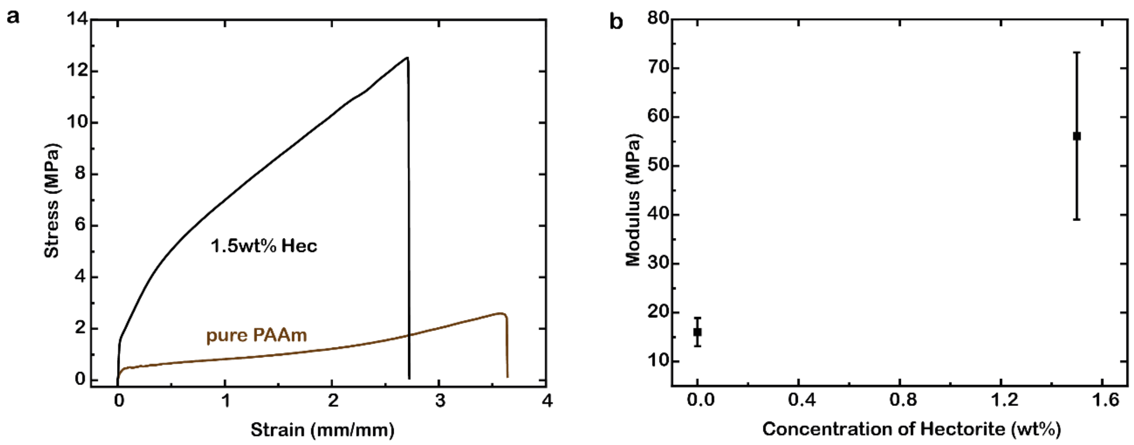

Supplementary Figure 29. Tensile tests of the Hec (1.5 wt%)-PAAm (62 wt%) organo-hydrogel using glycerol/water mixture solvent. (a) stress-strain curves. (b) Young's moduli with mean values ± standard deviations from 5 samples. Solvent mixture: 55 wt% of glycerol and 45 wt% of water.

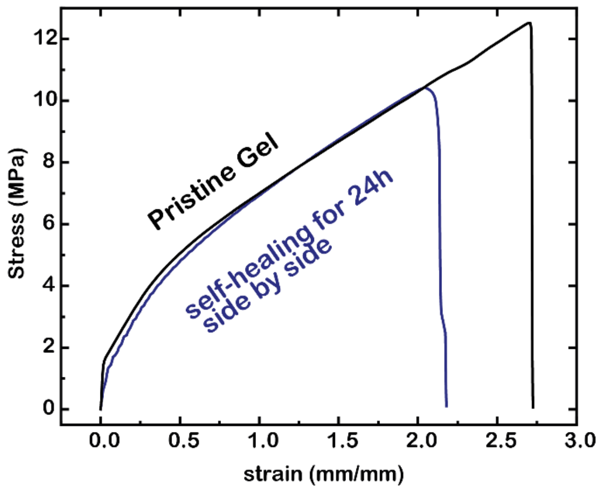

Supplementary Figure 30. Self-healing of Hec (1.5 wt%)-PAAm (62 wt%) organo-hydrogel in glycerol/water mixture solvent. The self-healing was conducted for 24 hours with a side-by-side configuration with an overlapped length of 2 mm. Solvent mixture: 55 wt% of glycerol and 45 wt% of water.

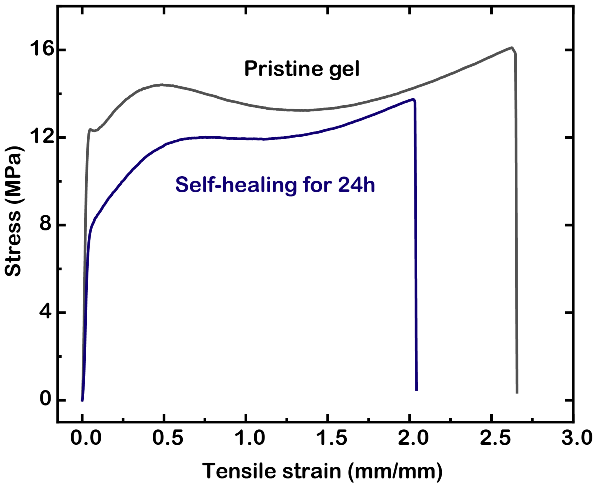

Supplementary Figure 31. Self-healing of Hec (2.8 wt%)-PAAm (65 wt%) organo-hydrogel in glycerol/water mixture solvent. The self-healing was conducted for 24 hours at 60 ℃ under side-by-side configuration with an overlapped length of 2 mm. Solvent mixture: 55 wt% of glycerol and 45 wt% of water.

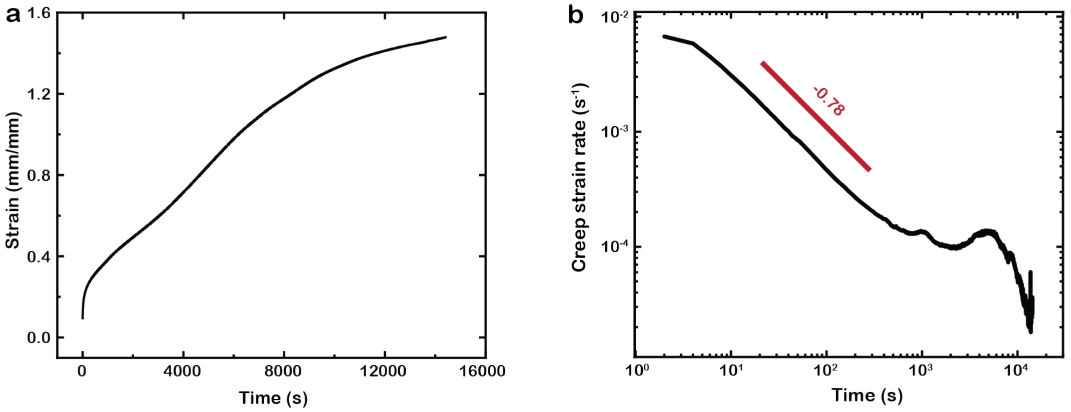

Supplementary Figure 32. Creep test of the Hec (1.5 wt%)-PAAm (62 wt%) organo-hydrogel under 1 MPa stress. (a) Creep strain with time. (b) Creep strain rate with time. Relative humidity during test: 50%. Solvent mixture: 55 wt% of glycerol and 45 wt% of water. The creep strain reaches 1.48 after 14 400 s under a constant stress of 1 MPa. The power law exponent of the creep strain rate relative to time is -0.78, similar to previous report on physically crosslinked hydrogels[63].

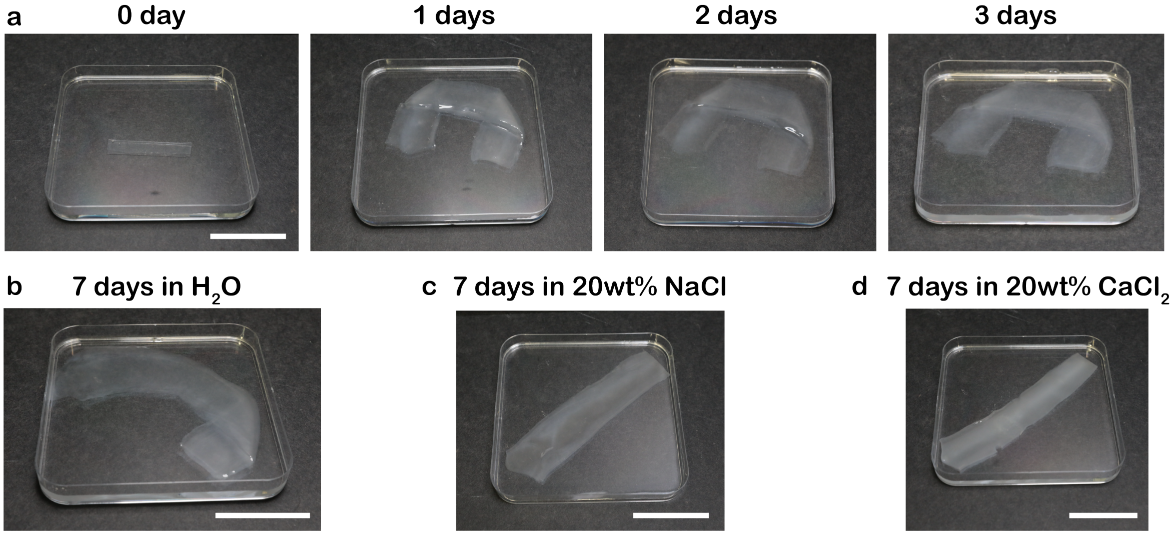

Supplementary Figure 33. Swelling stability of Hec-PAAm hydrogels at room temperature. (a) Photographs of Hec-PAAm hydrogel swelling in water. (b) Hec-PAAm hydrogel after swelling in water for 7 days. (c) Hec-PAAm hydrogel after swelling in 20 wt% NaCl aqueous solution for 7 days. (d) Hec-PAAm hydrogel after swelling in 20 wt% $CaCl_2$ aqueous solution for 7 days. The hydrogel contained 3 wt% of hectorite and 62 wt% of PAAm. Scale bars: 5 cm.

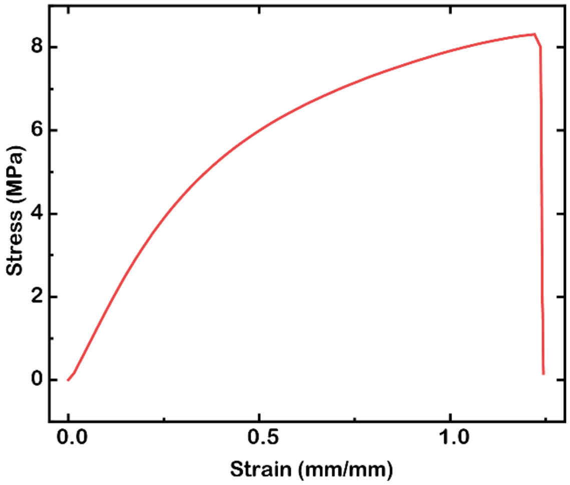

Supplementary Figure 34. Stress-strain curves of Fe^{3+} coordinated Hec (1.5 wt%)-PAAm (49.6 wt%)- PMAAc (12.4 wt%) hydrogel equilibrated in water for 2 days.

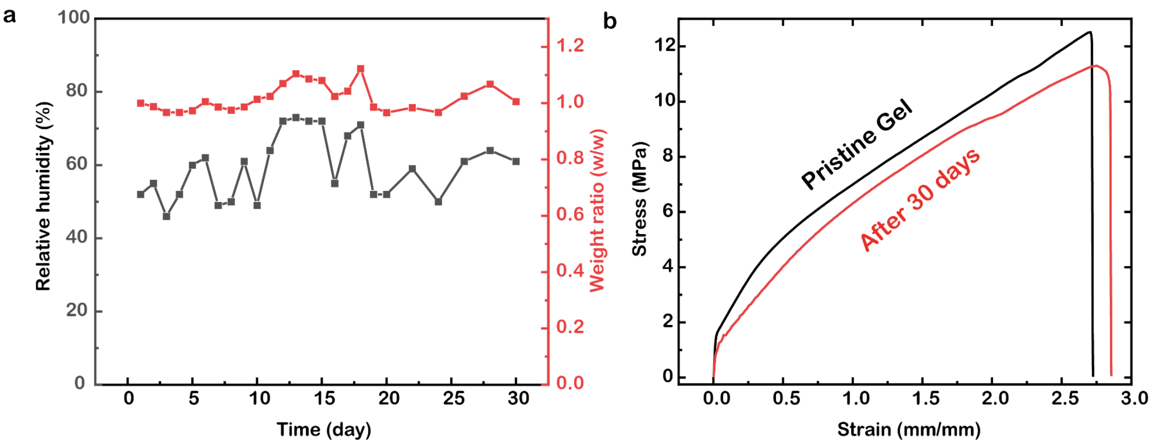

Supplementary Figure 35. Anti-dehydration test of the Hec (1.5 wt%) - PAAm (62 wt%) organo-hydrogel in glycerol/water mixture solvent. (a) Normalized weight change with corresponding relative humidity under ambient conditions for 30 days. (b) Stress-strain curve of the Hec (1.5 wt%)-PAAm (62 wt%) organo-hydrogel after storage for 30 days. Solvent mixture: 55 wt% of glycerol and 45 wt% of water.

5. Supplementary References

1. Aumiller, W. M., Cakmak, F. P., Davis, B. W. & Keating, C. D. RNA-Based Coacervates as a Model for Membraneless Organelles: Formation, Properties, and Interfacial Liposome Assembly. *Langmuir* **32**, 10042–10053 (2016).
2. François, J., Sarazin, D., Schwartz, T. & Weill, G. Polyacrylamide in water: molecular weight dependence of $\langle R^2 \rangle$ and [η] and the problem of the excluded volume exponent. *Polymer* **20**, 969–975 (1979).
3. Lide, D. R. (ed.) *CRC Handbook of Chemistry and Physics* (CRC Press, 2005).
4. Affdl, J. C. H. & Kardos, J. L. The Halpin-Tsai equations: A review. *Polym. Eng. Sci.* **16**, 344–352 (1976).
5. Kunz, D. A. *et al.* In-Plane Modulus of Singular 2:1 Clay Lamellae Applying a Simple Wrinkling Technique. *ACS Appl. Mater. Interfaces* **5**, 5851–5855 (2013).
6. Collins, D. R., Stirling, W. G., Catlow, C. R. A. & Rowbotham, G. Determination of acoustic phonon dispersion curves in layer silicates by inelastic neutron scattering and computer simulation techniques. *Phys. Chem. Miner.* **19**, (1993).
7. McNeil, L. E. & Grimsditch, M. Elastic moduli of muscovite mica. *J. Phys. Condens. Matter* **5**, 1681–1690 (1993).
8. Wan, S. *et al.* Strong sequentially bridged MXene sheets. *Proc. Natl. Acad. Sci.* **117**, 27154–27161 (2020).
9. Haraguchi, K., Uyama, K. & Tanimoto, H. Self-healing in Nanocomposite Hydrogels. *Macromol. Rapid Commun.* **32**, 1253–1258 (2011).
10. Liu, M. *et al.* An anisotropic hydrogel with electrostatic repulsion between cofacially aligned nanosheets. *Nature* **517**, 68–72 (2015).
11. Munier, P., Hadi, S. E., Segad, M. & Bergström, L. Rheo-SAXS study of shear-induced orientation and relaxation of cellulose nanocrystal and montmorillonite nanoplatelet dispersions. *Soft Matter* **18**, 390–396 (2022).
12. Röhrl, M. *et al.* Shear orientation of nematic phases of clay nanosheets: processing of barrier coatings. *J. Coat. Technol. Res.* **19**, 487–495 (2022).
13. Tang, Z., Kotov, N. A., Magonov, S. & Ozturk, B. Nanostructured artificial nacre. *Nat. Mater.* **2**, 413–418 (2003).

14. Yin, Z., Hannard, F. & Barthelat, F. Impact-resistant nacre-like transparent materials. *Science* **364**, 1260–1263 (2019).
15. Zhao, C. *et al.* Layered nanocomposites by shear-flow-induced alignment of nanosheets. *Nature* **580**, 210–215 (2020).
16. Wang, J., Lin, L., Cheng, Q. & Jiang, L. A Strong Bio-Inspired Layered PNIPAM-Clay Nanocomposite Hydrogel. *Angew. Chem. Int. Ed.* **51**, 4676–4680 (2012).
17. Ilyas, M. *et al.* Water-Triggered Ductile–Brittle Transition of Anisotropic Lamellar Hydrogels and Effect of Confinement on Polymer Dynamics. *Macromolecules* **50**, 8169–8177 (2017).
18. Aumiller, W. M., Pir Cakmak, F., Davis, B. W. & Keating, C. D. RNA-Based Coacervates as a Model for Membraneless Organelles: Formation, Properties, and Interfacial Liposome Assembly. *Langmuir* **32**, 10042–10053 (2016).
19. Shi, Y., Wu, B., Sun, S. & Wu, P. Aqueous spinning of robust, self-healable, and crack-resistant hydrogel microfibers enabled by hydrogen bond nanoconfinement. *Nat. Commun.* **14**, 1370 (2023).
20. Abdullah, T. & Okay, O. 4D Printing of Body Temperature-Responsive Hydrogels Based on Poly(acrylic acid) with Shape-Memory and Self-Healing Abilities. *ACS Appl. Bio Mater.* **6**, 703–711 (2023).
21. Jiang, Z. *et al.* Strong, Self-Healable, and Recyclable Visible-Light-Responsive Hydrogel Actuators. *Angew. Chem. Int. Ed.* **59***, 7049–7056 (2020).*
22. Shen, Z. *et al.* Low-water-content polyelectrolyte hydrogels inspired by human epidermal stratum corneum. *Cell Rep. Phys. Sci.* **4**, 101741 (2023).
23. Balavigneswaran, C. K. *et al.* Mussel-Inspired Adhesive Hydrogels Based on Laponite-Confined Dopamine Polymerization as a Transdermal Patch. *Biomacromolecules* **24**, 724–738 (2023).
24. Zheng, S. Y. *et al.* Metal-Coordination Complexes Mediated Physical Hydrogels with High Toughness, Stick–Slip Tearing Behavior, and Good Processability. *Macromolecules* **49**, 9637–9646 (2016).
25. Kang, J., Tok, J. B.-H. & Bao, Z. Self-healing soft electronics. *Nat. Electron.* **2**, 144–150 (2019).
26. Subraveti, S. N. & Raghavan, S. R. A Simple Way to Synthesize a Protective “Skin” around Any Hydrogel. *ACS Appl. Mater. Interfaces* **13**, 37645–37654 (2021).

27. Yuk, H., Zhang, T., Parada, G. A., Liu, X. & Zhao, X. Skin-inspired hydrogel–elastomer hybrids with robust interfaces and functional microstructures. *Nat. Commun.* **7**, 12028 (2016).
28. Yang, Y. *et al.* Biomimetic Porous MXene Sediment-Based Hydrogel for High-Performance and Multifunctional Electromagnetic Interference Shielding. *ACS Nano* **16**, 15042–15052 (2022).
29. Zhang, H. J. *et al.* Tough Physical Double-Network Hydrogels Based on Amphiphilic Triblock Copolymers. *Adv. Mater.* **28**, 4884–4890 (2016).
30. Luo, F. *et al.* Oppositely charged polyelectrolytes form tough, self-healing, and rebuildable hydrogels. *Adv. Mater.* **27**, 2722–2727 (2015).
31. Qin, H. *et al.* Dynamic Au-Thiolate Interaction Induced Rapid Self-Healing Nanocomposite Hydrogels with Remarkable Mechanical Behaviors. *Chem* **3**, 691–705 (2017).
32. Jiang, Z., Diggle, B., Shackleford, I. C. G. & Connal, L. A. Tough, Self-Healing Hydrogels Capable of Ultrafast Shape Changing. *Adv. Mater.* **31**, 1904956 (2019).
33. Qin, H., Zhang, T., Li, N., Cong, H. P. & Yu, S. H. Anisotropic and self-healing hydrogels with multi-responsive actuating capability. *Nat. Commun.* **10**, 1–11 (2019).
34. Sun, T. L. *et al.* Physical hydrogels composed of polyampholytes demonstrate high toughness and viscoelasticity. *Nat. Mater.* **12**, 932–937 (2013).
35. Fang, X. *et al.* Dynamic Hydrophobic Domains Enable the Fabrication of Mechanically Robust and Highly Elastic Poly(vinyl alcohol)-Based Hydrogels with Excellent Self-Healing Ability. *ACS Mater. Lett.* **2**, 764–770 (2020).
36. Ge, G. *et al.* $Ti_3C_2T_x$ MXene-Activated Fast Gelation of Stretchable and Self-Healing Hydrogels: A Molecular Approach. *ACS Nano* **15**, 2698–2706 (2021).
37. Caprioli, M. *et al.* 3D-printed self-healing hydrogels via Digital Light Processing. *Nat. Commun.* **12**, 2462 (2021).
38. Wang, Z. *et al.* A Rapidly Self-Healing Host-Guest Supramolecular Hydrogel with High Mechanical Strength and Excellent Biocompatibility. *Angew. Chem. Int. Ed.* **57**, 9008–9012 (2018).
39. Ding, Y. *et al.* High-Throughput Screening of Self-Healable Polysulfobetaine Hydrogels and their Applications in Flexible Electronics. *Adv. Funct. Mater.* **31**, 2100489 (2021).

40. Pei, X., Zhang, H., Zhou, Y., Zhou, L. & Fu, J. Stretchable, self-healing and tissue-adhesive zwitterionic hydrogels as strain sensors for wireless monitoring of organ motions. *Mater. Horiz.* **7**, 1872–1882 (2020).
41. Mo, J. *et al.* Design of ultra-stretchable, highly adhesive and self-healable hydrogels via tannic acid-enabled dynamic interactions. *Mater. Horiz.* **8**, 3409–3416 (2021).
42. Shao, C. *et al.* Mimicking Dynamic Adhesiveness and Strain-Stiffening Behavior of Biological Tissues in Tough and Self-Healable Cellulose Nanocomposite Hydrogels. *ACS Appl. Mater. Interfaces* **11**, 5885–5895 (2019).
43. Li, C.-H. *et al.* A highly stretchable autonomous self-healing elastomer. *Nat. Chem.* **8**, 618–624 (2016).
44. Kang, J. *et al.* Tough and Water-Insensitive Self-Healing Elastomer for Robust Electronic Skin. *Adv. Mater.* **30**, 1706846 (2018).
45. Zhu, X., Zhang, W., Lu, G., Zhao, H. & Wang, L. Ultrahigh Mechanical Strength and Robust Room-Temperature Self-Healing Properties of a Polyurethane–Graphene Oxide Network Resulting from Multiple Dynamic Bonds. *ACS Nano* **16**, 16724–16735 (2022).
46. Wang, Y., Huang, X. & Zhang, X. Ultrarobust, tough and highly stretchable self-healing materials based on cartilage-inspired noncovalent assembly nanostructure. *Nat. Commun.* **12**, 1291 (2021).
47. Chen, Y., Kushner, A. M., Williams, G. A. & Guan, Z. Multiphase design of autonomic self-healing thermoplastic elastomers. *Nat. Chem.* **4**, 467–472 (2012).
48. Lai, J.-C. *et al.* Thermodynamically stable whilst kinetically labile coordination bonds lead to strong and tough self-healing polymers. *Nat. Commun.* **10**, 1164 (2019).
49. Rao, Y.-L. *et al.* Stretchable Self-Healing Polymeric Dielectrics Cross-Linked Through Metal–Ligand Coordination. *J. Am. Chem. Soc.* **138**, 6020–6027 (2016).
50. Li, L. *et al.* High-Toughness and High-Strength Solvent-Free Linear Poly(ionic liquid) Elastomers. *Adv. Mater.* **36**, 2308547 (2024).
51. Lai, Y. *et al.* Colorless, Transparent, Robust, and Fast Scratch-Self-Healing Elastomers via a Phase-Locked Dynamic Bonds Design. *Adv. Mater.* **30**, 1802556 (2018).
52. Chen, H., Sun, Z., Lin, H., He, C. & Mao, D. Neuron Inspired All-Around Universal Telechelic Polyurea with High Stiffness, Excellent Crack Tolerance, Record-High Adhesion, Outstanding Triboelectricity, and AIE Fluorescence. *Adv. Funct. Mater.* **32**, 2204263 (2022).

53. Guo, H., Han, Y., Zhao, W., Yang, J. & Zhang, L. Universally autonomous self-healing elastomer with high stretchability. *Nat. Commun.* **11**, 2037 (2020).
54. Chen, L. *et al.* A Stiff yet Rapidly Self-Healable Elastomer in Harsh Aqueous Environments. *Adv. Funct. Mater.* **32**, 2107538 (2022).
55. Deng, Y., Zhang, Q., Feringa, B. L., Tian, H. & Qu, D.-H. Toughening a Self-Healable Supramolecular Polymer by Ionic Cluster-Enhanced Iron-Carboxylate Complexes. *Angew. Chem.* **132**, 5316–5321 (2020).
56. Cheng, Y. *et al.* A Fast Autonomous Healing Magnetic Elastomer for Instantly Recoverable, Modularly Programmable, and Thermorecyclable Soft Robots. *Adv. Funct. Mater.* **31**, 2101825 (2021).
57. Cao, P.-F. *et al.* Superstretchable, Self-Healing Polymeric Elastomers with Tunable Properties. *Adv. Funct. Mater.* **28**, 1800741 (2018).
58. Zhang, Y. *et al.* Super-Tough and Fast Adhesion of Soft Elastomer Based on Strong Noncovalent Interaction in Diverse Environments. *Adv. Funct. Mater.* **33**, 2304653 (2023).
59. Chen, L. *et al.* Facile synthesis of novel elastomers with tunable dynamics for toughness, self-healing and adhesion. *J. Mater. Chem. A* **7**, 17334–17344 (2019).
60. Zhang, Q. *et al.* Exploring a naturally tailored small molecule for stretchable, self-healing, and adhesive supramolecular polymers. *Sci. Adv.* **4**, eaat8192 (2018).
61. Chen, H. *et al.* 3D Printable, Biomimetic Adhesive, and Self-healing Acrylic Elastomers for Customized Attachable Strain Sensor. *Chem. Eng. J.* **430**, 133111 (2022).
62. Qu, X. *et al.* Solid-state and liquid-free elastomeric ionic conductors with autonomous self-healing ability. *Mater. Horiz.* **7**, 2994–3004 (2020).
63. Karobi, S. N. *et al.* Creep Behavior and Delayed Fracture of Tough Polyampholyte Hydrogels by Tensile Test. *Macromolecules* **49**, 5630–5636 (2016).
